# Genetic analysis of cassava brown streak disease root necrosis using image analysis and genome-wide association studies

**DOI:** 10.3389/fpls.2024.1360729

**Published:** 2024-03-18

**Authors:** Leah Nandudu, Christopher Strock, Alex Ogbonna, Robert Kawuki, Jean-Luc Jannink

**Affiliations:** ^1^ School of Integrative Plant Sciences, Section of Plant Breeding and Genetics, Cornell University, Ithaca, NY, United States; ^2^ Root Crops Department, National Crops Resources Research Institute (NaCRRI), Kampala, Uganda; ^3^ US Department of Agriculture, Agricultural Research Service (USDA-ARS), Ithaca, NY, United States

**Keywords:** root necrosis, image analysis, PlantCV, genome-wide association studies (GWAS), cassava brown streak disease (CBSD)

## Abstract

Cassava brown streak disease (CBSD) poses a substantial threat to food security. To address this challenge, we used PlantCV to extract CBSD root necrosis image traits from 320 clones, with an aim of identifying genomic regions through genome-wide association studies (GWAS) and candidate genes. Results revealed strong correlations among certain root necrosis image traits, such as necrotic area fraction and necrotic width fraction, as well as between the convex hull area of root necrosis and the percentage of necrosis. Low correlations were observed between CBSD scores obtained from the 1-5 scoring method and all root necrosis traits. Broad-sense heritability estimates of root necrosis image traits ranged from low to moderate, with the highest estimate of 0.42 observed for the percentage of necrosis, while narrow-sense heritability consistently remained low, ranging from 0.03 to 0.22. Leveraging data from 30,750 SNPs obtained through DArT genotyping, eight SNPs on chromosomes 1, 7, and 11 were identified and associated with both the ellipse eccentricity of root necrosis and the percentage of necrosis through GWAS. Candidate gene analysis in the 172.2kb region on the chromosome 1 revealed 24 potential genes with diverse functions, including ubiquitin-protein ligase, DNA-binding transcription factors, and RNA metabolism protein, among others. Despite our initial expectation that image analysis objectivity would yield better heritability estimates and stronger genomic associations than the 1-5 scoring method, the results were unexpectedly lower. Further research is needed to comprehensively understand the genetic basis of these traits and their relevance to cassava breeding and disease management.

## Introduction

Cassava (*Manihot esculenta* Crantz) is a vital tropical food crop, ranking third in caloric contribution after maize and rice. Its large starchy roots and edible leaves serve as a primary food source for more than 800 million people, primarily in sub-Saharan Africa (Nassar & Ortiz, 2010).

Over the past 90 years, cassava production has faced escalating threats from biotic and abiotic stresses (Tomlinson et al., 2018) exacerbated by climate change (Jarvis et al., 2012). Cassava brown streak disease (CBSD), ranked among the seven most serious threats to world food security (Pennisi, 2010), stands as a significant biotic challenge to cassava production, particularly in East, Central and Southern Africa, with the potential to cause losses of up to 100% in susceptible varieties (Hillocks et al., 2000; Hillocks & Jennings, 2003; Kaweesi et al., 2014). CBSD is caused by a positive sense single-stranded RNA virus belonging to the genus *Ipomovirus* and family *Potyviridae* (Winter et al., 2010; Walker et al., 2022). It is caused by two distinct viruses: cassava brown streak virus (CBSV) and Uganda cassava brown streak virus (UCBSV), both collectively referred to as cassava brown streak viruses (CBSVs). Both viruses are transmitted by whiteflies (*Bemisa tabaci*) in a semi-persistent manner (Maruthi et al., 2005; Mero et al., 2021) and through the movement of infected stem cuttings by farmers, which adds to the complexity of controlling the disease. CBSD symptoms are characterized by the initial emergence of leaf chlorosis along secondary vein margins, which later develop into blotches. Subsequently, brown streaks develop on the stem, accompanied by radial root constrictions and root necrosis (Hillocks & Jennings, 2003; Alicai et al., 2007; Kaweesi et al., 2014). The most destructive symptom is root necrosis, as it makes the cassava tubers unfit for consumption by both humans and animals. Therefore, it is essential to thoroughly investigate CBSD root necrosis to uncover the genetic markers or genetic mechanisms that can be leveraged in breeding for resistance. This can be comprehended by adopting root necrosis image capture and analysis to complement the 1-5 visual severity scores. This incorporation of root necrosis imaging could introduce fresh opportunities for enriching the qualitative 1-5 scoring approach with more precise quantitative analyses.

High-throughput phenotyping based on images holds enormous promise in unraveling the genetic basis of root necrosis. Image analysis enables the dissection of the genetic architecture of root necrosis by harnessing valuable information from variation in root necrosis expression patterns, which are often challenging to characterize with the naked eye (Demidchik et al., 2020). The effectiveness of image analysis for crop phenotyping has been showcased in a variety of species, including beans (Kumar et al., 2013; Sun et al., 2016), rice (Wu et al., 2019), potatoes (Cassells et al., 1999; Siebring et al., 2019). Its success extends to the study of diverse traits, ranging from plant architecture to chlorophyll content (Yadav et al., 2010; Dutta Gupta et al., 2013; Walter et al., 2015). Image analysis in plants has also been expanded to multiple pathosystems, enabling the detection and quantification of plant diseases (Sun et al., 2014; Elliott et al., 2022; Palma et al., 2022; Shi et al., 2023). Success of image analysis relies on a diverse range of image analysis tools, including Image J/Fuji (Schindelin et al., 2012, 2015, 2017), Brushlets (Meyer & Coifman, 1997), PlantCV (Gehan et al., 2017), and many others. Plant Computer Vision (PlantCV) is an open-source, high-throughput image analysis tool that can be used to create customized workflows for segmenting and measuring quantitative characteristics of plants from images (Gehan et al., 2017). PlantCV Version 2.1 is a Python-based package comprised of modular functions, which has remarkable flexibility, usability, and functionality in processing images from multiple platforms including red, green, and blue (RGB), Near-infrared (NIR), PSII fluorescence, thermal, and hyperspectral sensors. PlantCV has found application in the phenotypic characterization of a diversity of plant species including pennycress, *Arabidopsis*, wheat, teff, rice, common bean, quinoa, and more (Castillo et al., 2022; Knapp et al., 2022; Griffiths et al., 2023; Pierz et al., 2023), underscoring its significance as a valuable tool for quantitative classification of plant phenotypes.

Substantial advancements in next-generation sequencing (NGS) and statistical methodologies have opened new avenues for plant breeders to use state-of-the-art tools and methods to adopt more efficient strategies when developing improved cassava varieties (Wolfe et al., 2016). CBSD root necrosis is a trait in cassava for which the genetic architecture can be explored by leveraging DNA markers spread throughout the entire cassava genome. Integration of DNA markers has gained widespread acceptance in genome-wide association studies (GWAS) and has proven invaluable in revealing the extensive allelic diversity present within natural populations (Wang et al., 2020; Xiao et al., 2022). As a result, GWAS has enabled the identification of genomic regions or QTLs (Quantitative Trait Loci) associated with desirable traits that have been leveraged in implementing marker-assisted selection (MAS) and/or genomic selection (Rabbi et al., 2022). Quantitative root necrosis assessment using image analysis introduces novel traits that provide precise measurements and capture spatial characteristics overlooked by the traditional 1-5 scoring method, which relies on human observation to quantify root necrosis. To implement quantitative root necrosis assessments, Diversity Arrays Technology (DArT) markers, which detect thousands of genetic loci through microarray technology were used to conduct GWAS for root necrosis. These markers rely on hybridization of genomic DNA fragments to microarrays with probes for multiple loci, enabling detection of genetic polymorphisms such as SNPs and indels (Deres and Feyissa, 2023). Given that this marks the first attempt to investigate the genetic architecture of CBSD root necrosis using imaging. There is a necessity to identify genomic regions and alleles linked to these traits, potentially resulting in the discovery of novel genetic loci associated with CBSD resistance. This is because for more than 9 decades, CBSD evaluations have primarily relied on a qualitative 1-5 visual scoring scale (Tomlinson et al., 2018) which hinders the precise quantitative assessment of CBSD root necrosis, thus restricting the potential to reveal functional genomic insights. These insights can play a crucial role in utilizing marker-assisted selection or genomic selection to expedite genetic gains in breeding for CBSD root necrosis resistance.

In this study, we used data from PlantCV and CBSD severity scores to achieve four main objectives: (1) characterizing root necrosis image traits through broad-sense and narrow-sense heritability, genotype by trait (GT), and genotype plus genotype-by-environment interaction (GGE) biplots, (2) determining phenotypic and genetic correlations, (3) identifying genomic regions through univariate and multivariate GWAS analyses, and (4) categorizing candidate genes associated with root necrosis image traits and CBSD severity in the Cycle 2 population of genomic selection.

## Materials and methods

### Plant material and field conditions

The Cycle (C2) population of genomic selection, which consisted of 471 cassava clones used in this study, was developed at the National Crops Resources Research Institute (NaCRRI), Uganda (Nandudu et al., 2023). This population was developed through a series of selection and hybridization cycles involving clones selected based on genomic estimated breeding values (GEBVs) derived from the initial cycle (C0) and the first cycle (C1) populations. A comprehensive selection process identified 95 clones from the C1 population, which were then crossed, resulting in the creation of a population of 6,570 seedlings. From this pool, 471 cassava clones for the C2 population were selected. This C2 population was then evaluated in two clonal evaluation trials (CETs) that were planted in two locations (Namulonge and Serere) in 2019/2020 and 2020/2021. CETs were set up using an augmented incomplete block design, with three check varieties (UG110017, TME204 and Mkumba) planted in each block. Each plot consisted of ten plants, arranged in a single row with a spacing of 1m *1m. Additionally, spreader rows of a CBSD susceptible clone (TME 204) were planted around the experimental field and between blocks to enhance disease pressure.

### Root necrosis image acquisition

**Figure 1:**
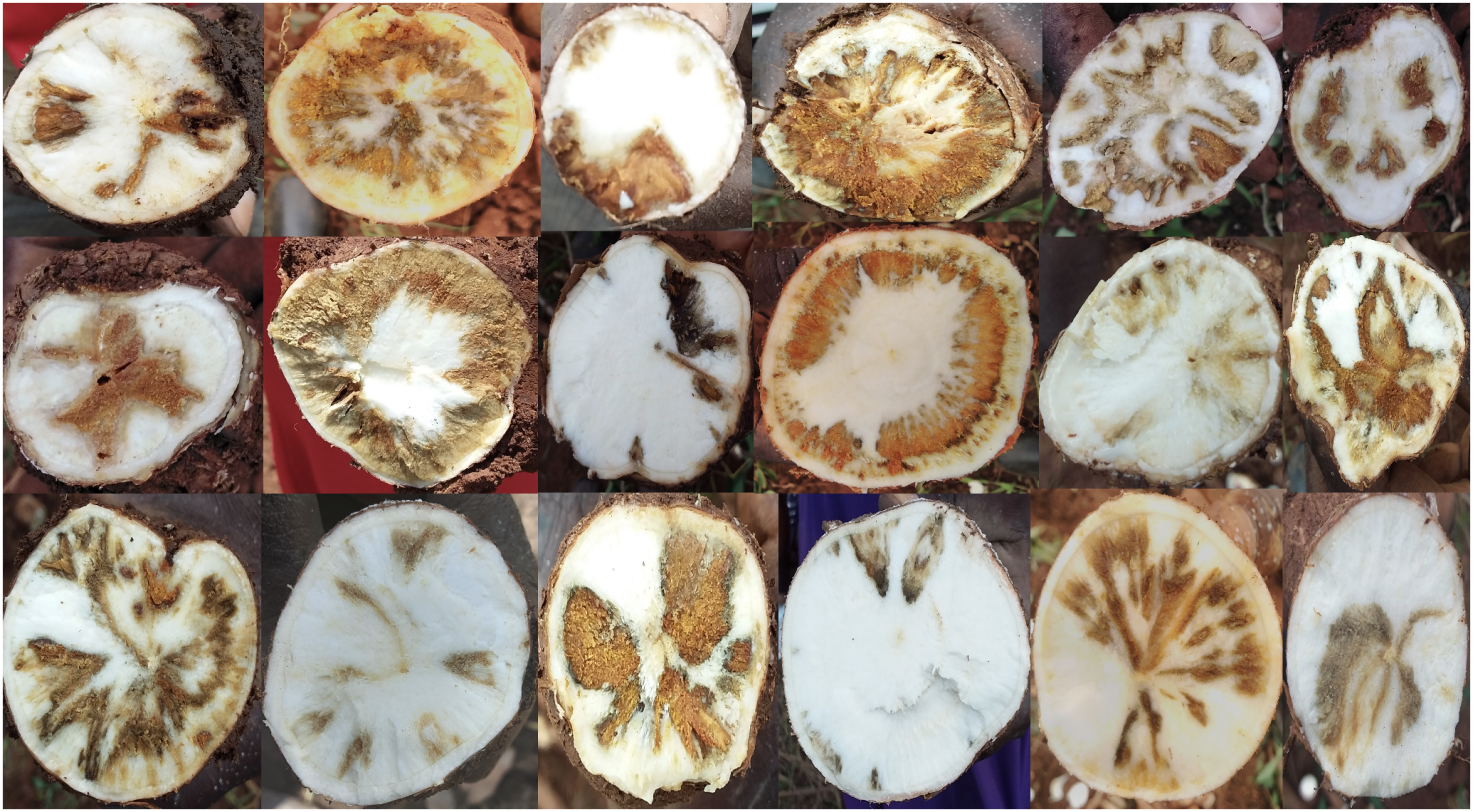
Examples of root necrosis manifestation as caused by cassava brown streak viruses in the C2 population.

During the harvest, five cassava roots were randomly sampled from each plant within a plot. The selection of only five roots was done to ensure consistency across all plots, considering that the number of cassava roots per plant varied. Subsequently, three images were captured by slicing each root at proximal, middle, and distal points as illustrated by (Ishmael and Emmanuel, 2020) using a sharp knife. In the field, RGB images were captured using Android tablets equipped with the Field Book App (Rife, 2016). This approach was employed to ensure the integrity of both the images and the associated data. 51,194 images (**Figure 1**) were collected from 471 clones. Note that we expected more images, but losses occurred due to natural calamities and poor sprouting of stem cuttings, particularly at the Serere experimental site. All images have been made accessible on Cassava base (Tecle et al., 2014; Morales et al., 2022) in Joint Photographic Experts Group (JPEG) formats.

### Root necrosis image processing

Root necrosis images underwent processing to measure root necrotic lesion traits from the root discs.

#### Original image

**Figure 2:**
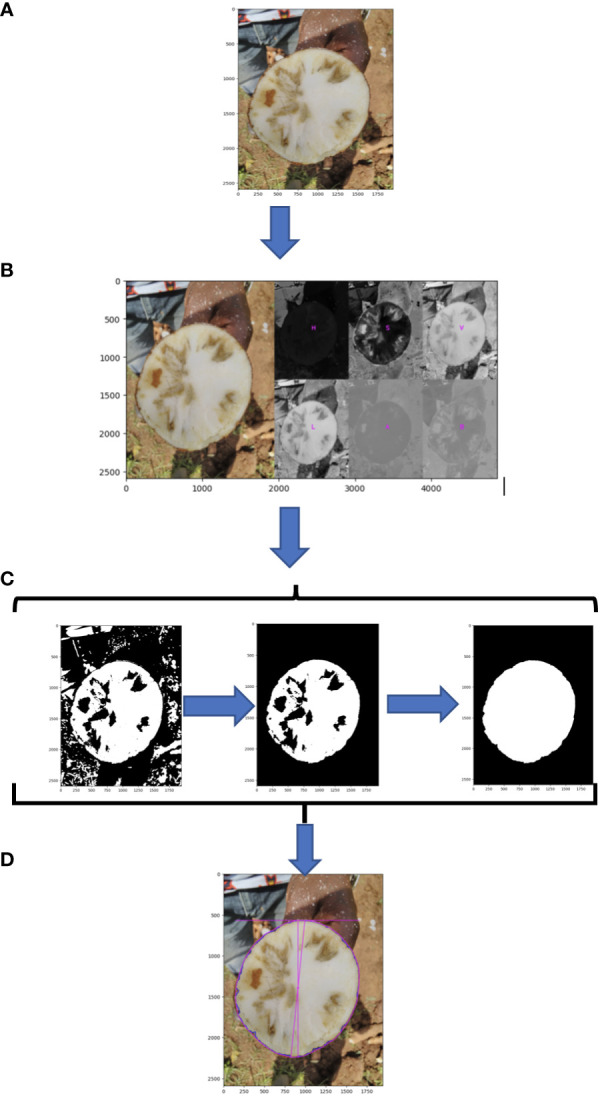
Schematic of root disc processing steps; **(A)** original image; **(B)** segmentation of root cross section using L channel from the CIELAB (L*a*b*) (lightness, green magenta, blue yellow) color space; **(C)** morphological image processing; **(D)** measure of total root cross-section.

Root necrosis images (**Figure 2A** and **Supplementary Figure 1A**) were labeled with an identification code containing the clone’s name, plot number, plant number, root number, and image position on the root.

#### Segmentation of root disc cross-section and root necrotic lesions

The original RGB image was transformed to HSV (Hue, saturation, value) color space to segregate the root disc from the background. To isolate necrotic lesions from the root disc, the RGB image was converted using the CIELAB (L*a*b*) (lightness, green magenta, blue, yellow) color space (**Figure 2B**). The L channel was then isolated, and the background was removed using the selection created in the root disc segmentation step. A default lower threshold of 160 and an upper threshold of 255 were used to analyze the L channel for the identification and selection of necrotic lesions. Subsequently, morphological image processing operations were employed in Python to eliminate background noise and fill holes in both the root cross-section and necrotic lesions, leaving them both as single and solid objects (**Figure 2C**, **Supplementary Figure 1B**, and source code).

#### Segmented image processing

Using PlantCV, various shape properties of both root cross-section and necrotic area were measured including: (1) total root cross-sectional area (**Figure 2D**), (2) convex hull area of the cross-section, (3) cross-section perimeter, (4) cross-section width, (5) cross-section height, (6) longest path across the centroid of the cross-section, (7) cross-section eccentricity, (8) cross-section major axis, and (9) cross-section minor axis. For the root cross section, we refer to these shape properties as “root1” to “root9”. Shape properties of the total necrotic regions were also measured including: (1) total necrotic area, (2) convex hull necrotic area, (3) necrotic perimeter, (4) necrotic width, (5) necrotic height, (6) necrotic longest path across the centroid, (7) necrotic ellipse eccentricity, (8) necrotic ellipse major axis, and (9) necrotic ellipse minor axis. For the necrotic area, we refer to these shape properties as “necro1” to “necro9”. All quantified traits were documented in pixels and subsequently normalized to those of the root disc, as outlined in **Table 1**. Only normalized traits were employed for subsequent analyses.

**Table 1:** Characterization and phenotypic variation of CBSD root necrosis traits in the C2 population.

	Trait Name	Acronyms	Trait description	Mean and standard deviation	Variance	Broad-sense heritability	Narrow-sense heritability
1	Solidity of necrosis	SN	Ratio of area of root necrosis to the convex hull area of root necrosis. SN = necro1/necro2.	0.29 ± 0.28	0.08	0.16	0.03
2	Convex hull area of root necrosis	CHAN	Area of the smallest convex polygon containing necrotic points. CHAN = necro2/root1.	3.03 ± 0.27	0.08	0.14	0.22
3	Ellipse eccentricity of root necrosis	EEN	Ratio of the distance of the focus from the center of the ellipse of the necrotic lesion and the distance of one end of the ellipse from the center. EEN = necro7.	0.79 ± 0.17	0.03	0.27	0.09
4	Percentage of necrosis	NECRO	Percentage area of the total root cross section that is necrotic. NECRO = necro1/root1.	0.69 ± 1.28	1.65	0.42	0.15
5	Necrotic area fraction	NAF	Ratio of the entire region of the root cross section affected by necrosis to Area of the smallest convex polygon containing necrotic points. NAF = necro2/root1.	-2.15 ± 1.80	3.25	0.23	0.17
6	Necrotic width fraction	NWF	Ratio of the widest part of the entire necrotic area to the width of the widest part of the root cross-section. NWF = necro4/root4.	-0.77 ± 0.98	0.95	0.21	0.13

### Phenotyping CBSD using the 1-5 scoring method

Disease severity at 3, 6 and 12 MAP were measured with the 1-5 visual scoring scale (Legg and Thresh, 1998). CBSD foliar severities were determined based on symptom expression on the leaves and stems for each plant in a plot. A score of 1 denoted no symptoms; 2 slight foliar chlorotic leaf mottling with no stem lesions; 3 foliar chlorotic leaf mottling and blotches with mild stem lesions, but no die back; 4 foliar chlorotic leaf mottling and blotches with pronounced stem lesions, but no die back; and 5 defoliation with stem lesions and dieback. Root severity scores were ascertained by visually evaluating the ratio of necrotic lesions to the total area of cross-sectioned root discs obtained from five roots per plant within a plot. In the root severity scoring, a score of 1 indicated absence of necrosis, 2 represented less than 5% necrotic lesions; 3 denoted 6 – 10% necrotic lesions; 4 signified 11 – 25% necrotic lesions and mild root constriction; while 5 denoted >25% necrotic lesions with severe root constriction.

### DArTseq genotyping

Of the 471 clones in the CET, 320 were chosen at random for genotyping. For each clone, two young top leaves were collected, folded, and punched using a 5mm hand puncher before being placed in 96-well plates. Subsequently, DNA extraction, Genotyping-by-Sequencing, and SNP calling were performed for each sample using the DArTseq genotyping platform (https://www.diversityarrays.com/technology-and-resources/dartreseq/). 28,434 markers were identified through DArTseq, and these were combined with an additional set of GBS-imputed genotypic data for the same lines (Marnin Wolfe, unpublished data) resulting in a dataset containing 51,860 SNPs. The integration of both marker datasets improved SNP coverage. To enhance the association power and address potential sequencing errors, an additional filtering step was applied to the combined marker dataset. Genotypes with more than 10% missing data and SNPs with over 5% missing data or minor allele frequency below 5% were removed. After filtering, a total of 30,750 SNP markers were obtained and used for downstream analyses.

### Statistical analyses

#### Descriptive statistical measures describing root necrosis image traits

The root necrosis image data displayed significant skewness and non-normal distributions. To address this, most traits underwent natural logarithm transformation, except for solidity of necrosis, and necrotic ellipse eccentricity, which remained unchanged. Also, to visualize the relationships among clones in the C2 population and evaluate their performance across the two environments, the dataset containing root necrosis image traits underwent analysis using both genotype by trait (GT) and genotype plus genotype-versus-environment interaction (GGE) biplot analyses. GT and GGE analyses were executed via the *gtb* and *gge* functions respectively within the R package *metan* (Olivoto and Lúcio, 2020). These functions used the first two principal components (PC) extracted through singular value decomposition (SVD) to partition the standardized genotype by trait and genotype by environment tables, yielding eigenvalues for genotypes, traits, environments, and singular values. In the biplot, the cosine of the angle between the vectors of two traits approximated the Pearson correlation with an angle less than 90° indicating a positive correlation, greater than 90° indicating a negative correlation and 90° indicating a zero correlation (Ma et al., 2004; Yan and Frégeau-Reid, 2018). Descriptive statistics of mean, standard deviation and variance were also calculated using base functions in R.

#### Phenotypic and genetic correlations

Phenotypic and genetic correlations were calculated for both CBSD root necrosis image traits and severity scores from the 1-5 visual scoring scale. These were computed using log-transformed phenotypic values and deregressed Best linear unbiased predictions (BLUPs). Correlation analyses were performed using the *cor* function in the R package (R Development Core Team, 2016), and visualization of the correlation matrices was done using the ‘*corrplot*’ R package (Wei and Simko, 2017).

#### Broad-sense and narrow-sense heritability

A standard linear mixed effects model was used to partition variance components using the *lme4* package in R (R Development Core Team, 2016) to determine broad-sense heritability.

A mixed linear model for the observed root necrosis image traits was fitted.


$$\text{y}=\text{Xb}+{\text{Z}}_{\text{c}}\text{c}+{\text{Z}}_{\text{b}}\text{B}+{\text{Z}}_{\text{p}}\text{p}+{\text{Z}}_{\text{n}}\text{n}+{\text{Z}}_{\text{r}}\text{r}+\epsilon$$

where **y** represents the vector of phenotypic data related to root necrosis image traits, while X denotes an n×q known design matrix encompassing fixed effects for location, year, checks, and image number. The vector b represents the coefficients associated with these fixed effects.

Z_c,_ Z_b_, Z_p_, Z_n_ and Z_r_ are known design matrices of random effects clone ID, block effects, plot ID, plant ID, and root ID with distributions of c ~ N (0, Iσ_c_^2^), B ~ N (0, Iσ_B_^2^), p ~ N (0, Iσ_p_^2^), n ~ N (0, Iσ_n_^2^), and r ~ N (0, Iσ_r_^2^). ϵ is the residual with a distribution of ϵ ~N (0, Iσ_e_^2^).

Broad-sense heritability was determined using H^2^ = σ _c_^2^ / [σ _c_^2^ + σ_P_^2^/n_P_ + σ_N_^2^/n_N_ + σ_R_^2^/n_R_ + σ _ϵ_^2^/n_I_] Where σ**_c_** ^2^ is the genotypic variance, σ_P_^2^ plot variance, σ_N_^2^ variance of plants within a plot, σ_R_^2^ variance of roots within a plant, and σ _ϵ_^2^ is the residual variance. The values n_P,_ n_N,_ n_R_ and n_I_ are the harmonic means the number of plots per clone, number of plants per clone, number of roots per clone, and number of images per clone. Best linear unbiased predictions (BLUPs) were obtained from the mixed linear model, and these were deregressed using the following equation:


$$deregressed\;BLUP=\frac{BLUP}{1-\frac{PEV}{\sigma_{c}^{2}}}$$

Where PEV was the prediction error variance of the BLUP and σ_c_^2^ variances of the genotypes (Garrick et al., 2009). Deregressed BLUPs were used for downstream analyses.

Narrow sense heritability was estimated using the function emmreml in the *EMMREML* package (Akdemir & Okeke, 2015) in R.

### Genome-wide association studies

Deregressed BLUPs of the 320 clones with genomic data for both root necrosis image traits and CBSD severity scores from the 1-5 scoring method (de los Campos et al., 2013) were used to perform univariate and multivariate GWAS using GEMMA version 0.98.4 with default parameters (Zhou, 2012; Zhou and Stephens, 2014). Univariate GWAS analysis was based on model; **Y = Wα+ x ***β*** + *U* +**ℇ**, *U* ~ MVN_n_ (0, λτ^-1^K),** ℇ **~ MVN_n_(0, τ^-1^I_n_)** where Y is an n-vector of CBSD root necrosis image traits and severity scores from the 1-5 scoring method; W is an n x c matrix of principal components as covariates including a column of 1s; α is a c-vector of the corresponding coefficients including the intercept; x is an n-vector of marker genotypes; β is the effect size of the marker; *U* is an n-vector of random effects; ℇ is an n-vector of errors; τ^-1^ is the variance of the residual errors; λ is the ratio between the random effect and error variances; K is a known n × n relatedness matrix and I_n_ is an n x n identity matrix. Using the *prcomp* function in R, principal components (PCs) were determined, and four (PCs) these were used to account for population structure.

Multivariate GWAS analysis was based on the model **Y =WA + x ***β*** ^t^ + *U* +** ℇ**, U ∼ MN_n×d_(**0**, K, V_g_),** ℇ **∼ MN_n×d_(**0**, I_n×n_, V_e_)** where Y is an *n* x *d* matrix of *d* CBSD root necrosis image traits phenotypes for *n* individuals; W is an *n×c* matrix of principal components as covariates including a column of 1s; A is a *c* by *d* matrix of the corresponding coefficients including the intercept; x is an n-vector of marker genotypes; β is a d vector of marker effect sizes for the d phenotypes; U is an n by d matrix of random effects; ℇ is an n by d matrix of errors; K is a known n by n relatedness matrix, I_n×n_ is a n by n identity matrix, V_g_ is a d by d symmetric matrix of genetic variance components, V_e_ is a d by d symmetric matrix of environmental variance components and MN_n×d_(0, V_1_, V_2_) denotes the n×d matrix normal distribution with mean 0, row covariance matrix V_1_ (n by n), and column covariance matrix V_2_ (d by d). Given the extensive number of traits in this study and our aim to enhance the likelihood of identifying shared candidate genes, we performed multivariate GWAS only on traits exhibiting high phenotypic and genotypic correlations. These trait combinations included: (1) convex hull area of root necrosis, percentage of necrosis, necrotic area fraction and necrotic width fraction, (2) necrotic area fraction and necrotic width fraction, (3) ellipse eccentricity of root necrosis, percentage of necrosis, necrotic area fraction and necrotic width fraction, (4) percentage of necrosis, necrotic area fraction and necrotic width fraction, (5) percentage of necrosis, necrotic area fraction and CBSD severity at 12 months after planting (CBSDS12). Despite the absence of strong phenotypic correlations, we conducted multivariate GWAS analyses for the necrosis percentage and CBSD severity at 3 months after planting (CBSDS3) as well as at 6 months after planting (CBSDS6). Additionally, a multivariate GWAS analysis was performed for the necrosis percentage and CBSD severity at 12 months after planting (CBSDS12). Visualization of Manhattan and quantile-quantile plots was carried out using the “*qqman*” R package (Turner and Turner, 2021). To account for multiple testing, the significance threshold was set at a corrected p-value of 0.05 divided by the number of markers on each chromosome, as described by Li and Ji (2005).

### Candidate gene analysis

Significant SNP markers linked to both root necrosis image traits and CBSD severity scores were used to determine genomic regions that were characterized for candidate genes. Gene positions were established using *M. esculenta* genome version 6, and any genes that overlapped with these significant genomic regions were classified as candidate genes. BEDTools were employed to detect potential genes regions where the significant SNPs were identified (Quinlan & Hall, 2010). Identified genes were characterized for gene ontology including molecular and biological functioning using PANTHER version 17.0 (Mi et al., 2019) and *M. esculenta* genome version 6 gene ontology database in Phytozome (Goodstein et al., 2012).

## Results

### Characterization of CBSD root necrosis image traits in the C2 population

**Figure 3:**
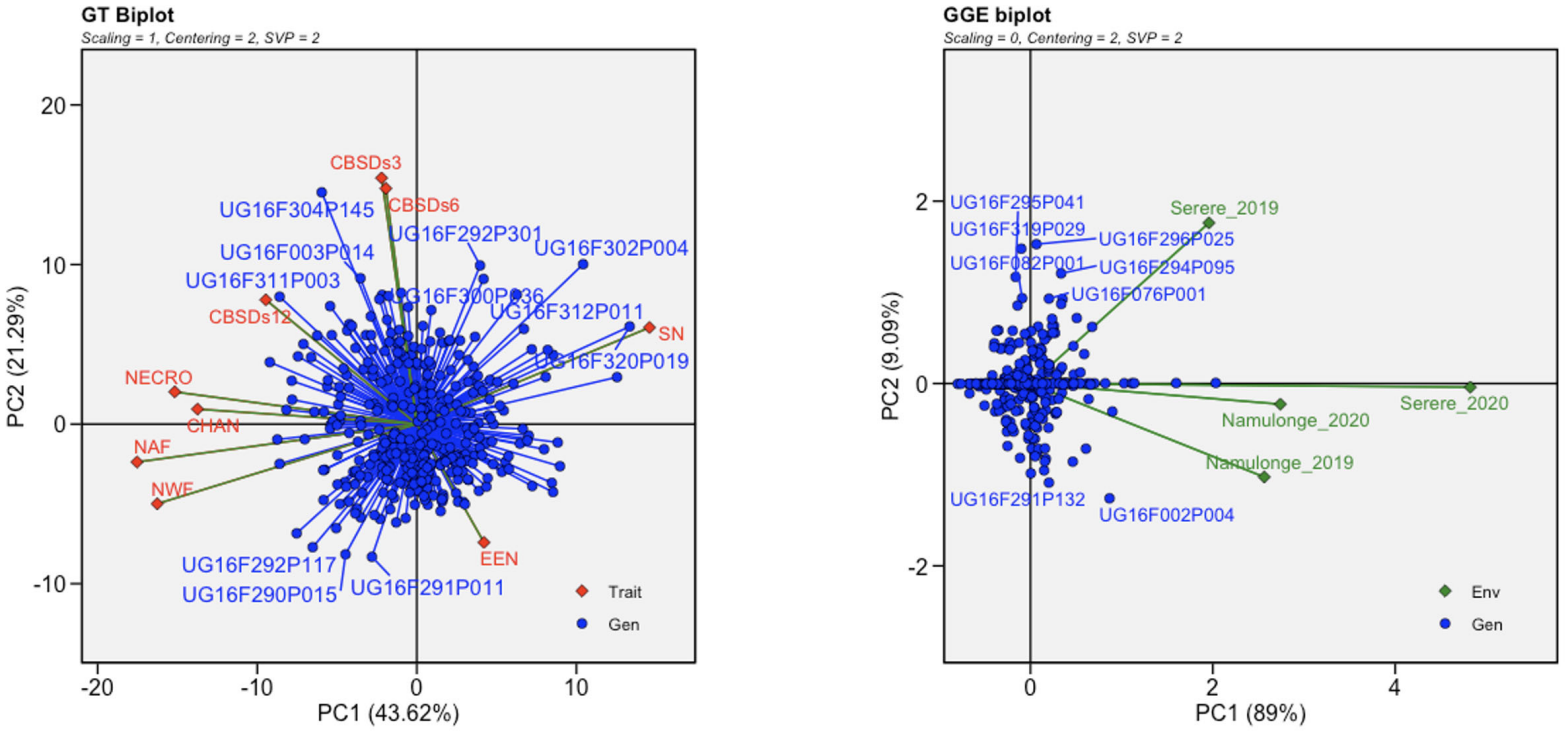
Genotype by trait (GT) and genotypic main effect plus genotype by environment interaction (GGE) biplots based on transformed root necrosis image data. GT trait codes are SD, Solidity of necrosis; CHAN, Convex hull area of root necrosis; EEN, Ellipse eccentricity of root necrosis; NECRO, Percentage of necrosis; NAF, Necrotic area fraction; NWF, Necrotic width fraction; CBSDs3, CBSD foliar severity at 3 MAP; CBSDs6, CBSD foliar severity at 6; CBSDs12, CBSD root severity at 12 MAP while GGE biplot shows the location-year scaled.

To visualize the relationships among genotypes, traits, and environments associated to both root necrosis image traits and the 1-5 visual severity scores, GT and GGE biplots were generated. Specifically, the convex hull area of root necrosis, necrotic area fraction, necrotic width fraction, and the percentage of necrosis exhibited strong positive correlations among themselves. Conversely, all these root necrosis image traits exhibited negative correlations with both the ellipse eccentricity of root necrosis and the solidity of necrosis. Interestingly, the root necrosis image traits, including convex hull area of root necrosis, necrotic area fraction, necrotic width fraction, and the percentage of necrosis, demonstrated weak but positive correlations with CBSDs3, CBSDs6, and CBSDs12, as shown in **Figure 3**. In contrast, ellipse eccentricity of root necrosis and the solidity of necrosis exhibited strong negative correlations. The GGE biplots revealed a positive correlation between datasets from the Namulonge experimental site for both years and the Serere experimental site in the 2020/2021 season. The dataset from the Serere experimental site in the 2019/2020 season was not correlated with all the other experimental sites. Moreover, within the C2 population, most clones displayed stability across different environments, with only a few exceptions. Notably, outliers such as UG16F002P004 and UG16F291P132 were observed in the Namulonge experimental site for both years, while clones UG16F07P001, UG16F295P041, UG16FF294P095, UG16F296P025, UG16F082P001, and UG16F319P029 exhibited unique characteristics in the Serere 2019/2020 experimental site.

Broad-sense heritability estimates for root necrosis image traits consistently spanned from low to moderate values (ranging between 0.14 and 0.42). Among these traits, the percentage of necrosis displayed the highest heritability estimate at 0.42, whereas the solidity of necrosis and the convex hull area of root necrosis exhibited the lowest broad-sense heritability estimates, measuring 0.16 and 0.14, respectively (**Table 1**). Regarding the CBSD severity scores derived from the 1-5 scoring method, the broad-sense heritability estimates stood at 0.73, 0.74, and 0.71 for CBSDs3, CBSDs6, and CBSDs12, respectively. Narrow-sense heritability estimates consistently remained low, ranging from 0.03 to 0.22. Among these estimates, the convex hull area of root necrosis exhibited the highest value at 0.22, while the solidity of necrosis and ellipse eccentricity of root necrosis displayed the lowest narrow-sense heritability estimates, measuring 0.03 and 0.09, respectively (**Table 1**). The percentage of necrosis was estimated at 0.15, whereas necrotic area fraction and necrotic width fraction had estimates of 0.17 and 0.13, respectively. In the context of CBSD severity scores obtained from the 1-5 scoring method, the narrow-sense heritability estimates were 0.34, 0.42, and 0.50 for CBSDs3, CBSDs6, and CBSDs12, respectively.

### Phenotypic and genetic correlations between root necrosis image traits and severity scores from 1-5 scoring method

**Figure 4:**
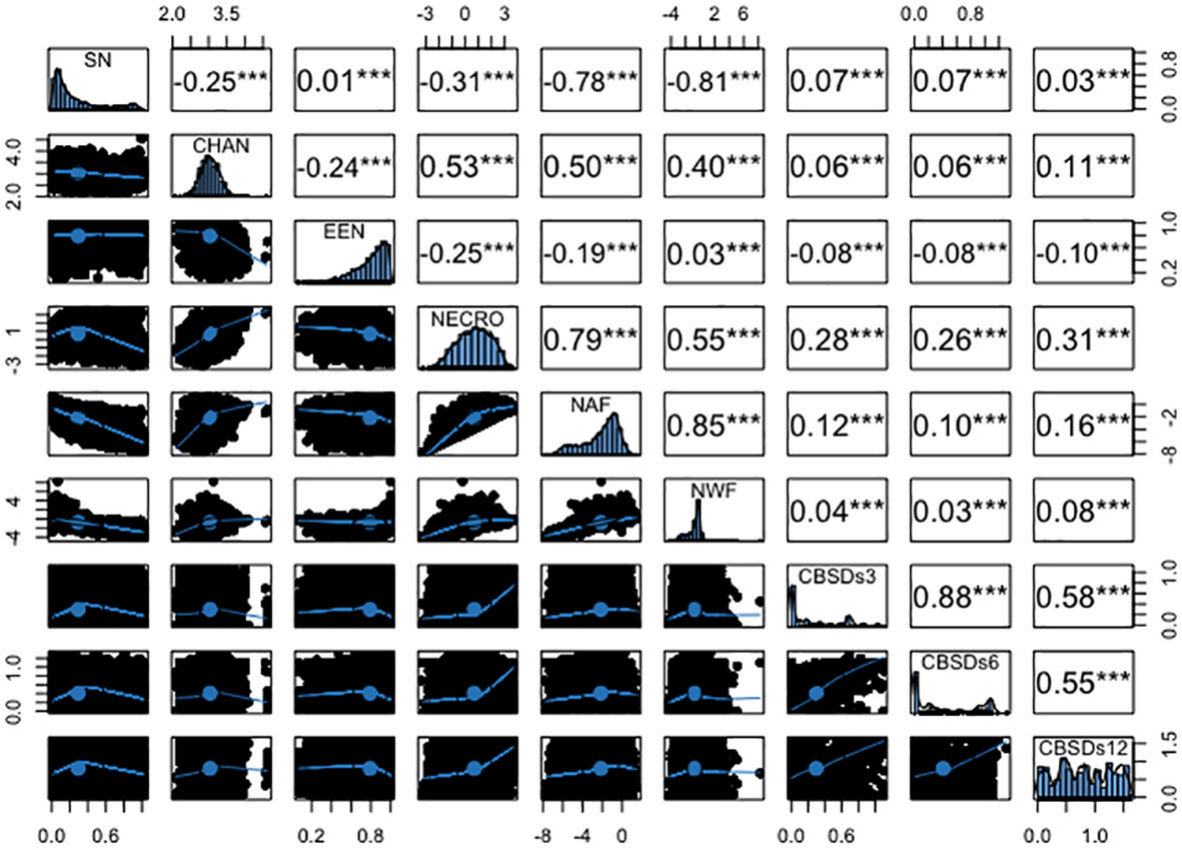
Phenotypic correlation coefficients among root necrosis imaging traits and visual scores from the 1-5 scoring method. Frequency distribution histograms are shown on the diagonal. SD, Solidity of necrosis; CHAN, Convex hull area of root necrosis; EEN, Ellipse eccentricity of root necrosis; NECRO, Percentage of necrosis; NAF, Necrotic area fraction; NWF, Necrotic width fraction; CBSDs3, CBSD foliar severity at 3 MAP; CBSDs6, CBSD foliar severity at 6MAP; CBSDs12, CBSD root severity at 12 MAP. Significant thresholds: *, P<0.05; **, P<0.01; ***, P<0.001.

The analysis revealed several significant correlations among root necrosis image traits, as shown in **Figure 4**. Notably, the necrotic area fraction and necrotic width fraction exhibited the strongest correlation at 0.85. Additionally, the convex hull area of root necrosis correlated positively with the percentage of necrosis (r = 0.53), necrotic area fraction (r = 0.50), and necrotic width fraction (r = 0.40). A strong correlation was also found between the percentage of necrosis and both necrotic area fraction (r = 0.79) and necrotic width fraction (r = 0.55). Low but positive phenotypic correlations were observed between the solidity of necrosis and the ellipse eccentricity of root necrosis (r = 0.07). In contrast, negative correlations ranged from -0.25 to -0.81 among solidity of necrosis, convex hull area of root necrosis, percentage of necrosis, necrotic area fraction, and necrotic width fraction. Moreover, ellipse eccentricity of root necrosis showed negative correlations with convex hull area of root necrosis, percentage of necrosis, and necrotic area fraction. The correlation coefficients between root necrosis image traits and severity scores from the 1-5 scoring method obtained at 3, 6, and 12 months after planting (MAP) were relatively low, ranging from -0.08 to 0.31. Notably, positive but modest correlation coefficients (ranging from -0.08 to 0.26) were observed between CBSDs3 and CBSDs6 and all root necrosis image traits. In the case of CBSDs12, correlation coefficients with root necrosis traits ranged from -0.10 to 0.31, with the percentage of necrosis demonstrating the highest correlation coefficient of 0.31. Interestingly, ellipse eccentricity of root necrosis consistently exhibited negative correlation coefficients with all the CBSD severity scores. Furthermore, correlations were evident within the CBSD severity scores themselves, including a strong correlation of r = 0.88 between CBSDs3 and CBSDs6. Additionally, moderately positive correlations (r = 0.58 and 0.55) were identified between CBSDs12 and CBSDs3, as well as between CBSDs12 and CBSDs6, respectively.

**Figure 5:**
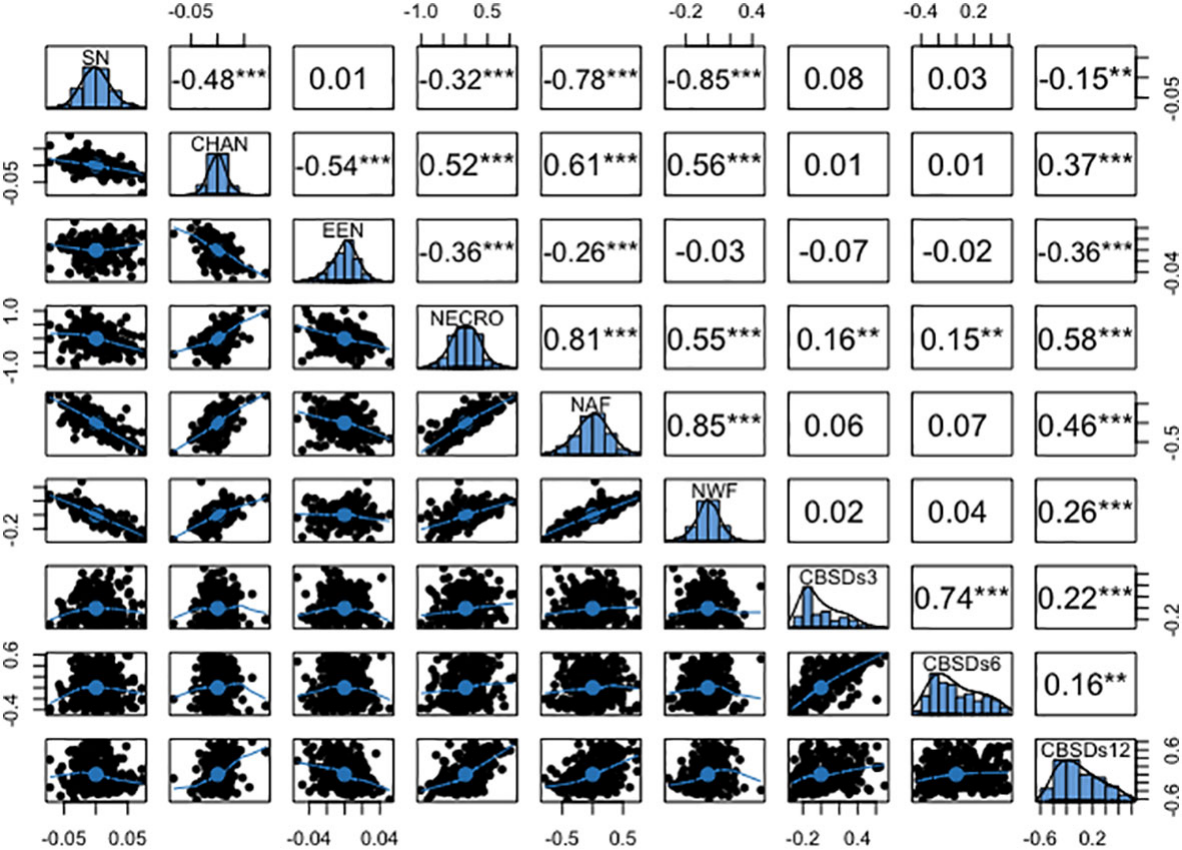
Genetic correlation coefficients among root necrosis imaging traits and visual scores from the 1-5 scoring method. Frequency distribution histograms are shown on the diagonal. SD, Solidity of necrosis; CHAN, Convex hull area of root necrosis; EEN, Ellipse eccentricity of root necrosis; NECRO, Percentage of necrosis; NAF, Necrotic area fraction; NWF, Necrotic width fraction; CBSDs3, CBSD foliar severity at 3 MAP; CBSDs6, CBSD foliar severity at 6 MAP; CBSDs12, CBSD root severity at 12 MAP. Significant thresholds: *, P<0.05; **, P<0.01; ***, P<0.001.

The genotypic correlations mirrored the pattern observed in the phenotypic correlations, as illustrated in **Figure 5**. Notably, a high genetic correlation emerged between necrotic area fraction and necrotic width fraction (r = 0.85), along with a similarly strong association between the percentage of necrosis and necrotic area fraction (r = 0.81). Moderate yet positive genetic correlations were observed between the convex area of root necrosis and the percentage of necrosis (r = 0.52), necrotic area fraction (r = 0.61), and necrotic width fraction (r = 0.56). Conversely, negative genetic correlations ranging from -0.03 to -0.85 were consistently observed between the solidity of necrosis and the eccentricity of root necrosis, as well as with all other root necrosis traits. In terms of genetic correlations between root necrosis traits and CBSD severity scores on the 1-5 scale, they spanned from low to moderate (r = 0.01 – 0.58). The percentage of necrosis (r = 0.58), necrotic area fraction (r = 0.46), and the convex hull area of root necrosis (0.37) exhibited the most substantial correlation coefficients with CBSDs12. However, correlation coefficients between CBSDs3 and CBSDs6 and most root necrosis traits failed to reach significance at the p-value threshold of 0.001. Correlations were also evident among the CBSD severity scores themselves, including a strong correlation of r = 0.74 between CBSDs3 and CBSDs6. Additionally, moderately positive correlations (r = 0.22 and 0.16) were identified between CBSDs12 and CBSDs3, as well as between CBSDs12 and CBSDs6, respectively.

### GWAS of CBSD traits in the C2 population

**Figure 6:**
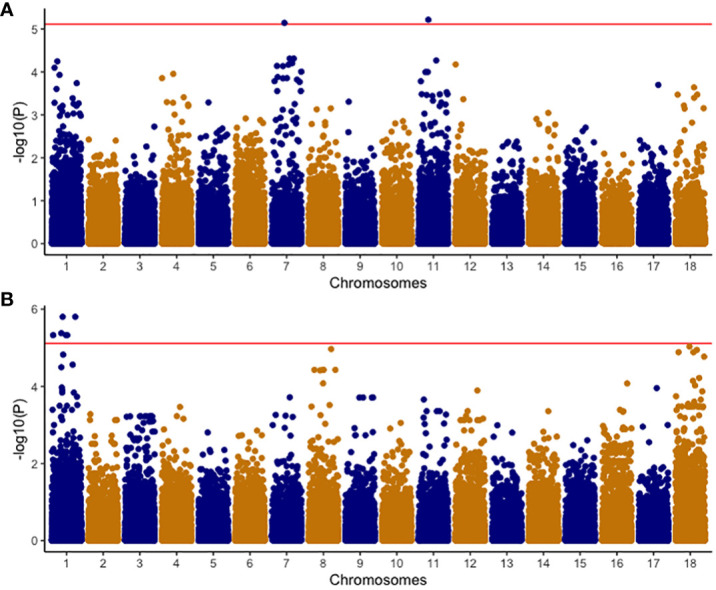
Manhattan plots of -Log_10_(P) showing chromosomal positions of SNP markers in univariate GWAS associated with **(A)** EEN; Ellipse eccentricity of root necrosis, and **(B)** NECRO; Percentage of necrosis. The red line represents the significant threshold -Log_10_ (P) value of 5.1 which was determined by using the effective number of independent tests on each chromosome to modify the Bonferroni correction method.

A comprehensive analysis was conducted on 320 clones within the C2 population, using both univariate and multivariate GWAS methodologies. In the univariate GWAS analysis, a total of eight SNPs were discovered across chromosomes 1, 7, and 11. These SNPs were determined to be linked with both the ellipse eccentricity of root necrosis and the percentage of root necrosis (**Table 2**, **Figure 6**, **Supplementary Figure 2**). Specifically, two SNPs on chromosomes 7 and 11 were linked to the ellipse eccentricity of root necrosis, while six SNPs on chromosome 1 were associated with the percentage of necrosis. Each significant marker accounted for a relatively small proportion of phenotypic variance, ranging from 0.01 to 0.41. Particularly, SNP markers S1_29063012 and S1_29103386 associated to the percentage of root necrosis were found to explain the highest variance among them (**Table 2**). No significant associations were observed with the other root necrosis image traits (**Supplementary Figures 3** and **4**). Concerning the CBSD severity scores, one SNP marker located on chromosome 1 was found to be associated with CBSDs12, whereas no SNPs displayed associations with CBSDs3 and CBSDs6 (**Supplementary Figures 5** and **6**). In the multivariate GWAS analysis, no significant associations were detected for various trait combinations, except for ellipse eccentricity of root necrosis, percentage of root necrosis, necrotic area fraction, and necrotic width fraction, where one SNP on chromosome 11 was identified (**Supplementary Figures 7**-**10**). This SNP, named S11_31660720, was also identified as associated with the ellipse eccentricity of root necrosis in the univariate GWAS analysis.

**Table 2:** Significant SNPs associated to Ellipse eccentricity of root necrosis and Percentage of necrosis and the phenotypic variance explained.

Trait	SNP	Chromosome	Position	P-value	PVE (%)
Ellipse eccentricity of root necrosis	S7_718516	7	718516	5.14	0.07
	S11_31660720	11	31660720	5.22	0.01
Percentage of necrosis	S1_28931143	1	28931143	5.32	0.40
	S1_28972056	1	28972056	5.32	0.40
	S1_28977687	1	28977687	5.32	0.40
	S1_29058977	1	29058977	5.80	0.32
	S1_29063012	1	29063012	5.38	0.41
	S1_29103386	1	29103386	5.80	0.41

### Candidate gene identification

Since most of the identified SNPs were located on chromosome 1, a region spanning 172.2kb was examined, leading to the identification of 24 potential candidate genes (**Supplementary Table 1**). These genes comprised a broad spectrum of functional annotations, including roles such as ubiquitin-protein ligase, ribosomal protein, transmembrane signal receptor, DNA-binding transcription factor, and RNA metabolism protein, among others. Molecular characterizations of these candidate genes suggest their involvement in catalytic activity (GO:0003824), binding (GO:0005488), and transcription regulator activity (GO:0140110) (**Supplementary Table 2**). Biological classifications of these potential candidate genes underscore their involvement in a wide array of processes, such as cellular processes (GO:0009987), metabolic processes (GO:0008152), biological regulation (GO:0065007), and localization (GO:0051179) (**Supplementary Table 3**).

## Discussion

Using PlantCV to extract root necrosis image traits and the 1-5 visual scoring method for foliar and root severities, we conducted a comprehensive characterization of cassava brown streak disease. Our aim was to identify genomic regions associated with CBSD root necrosis image and severity traits through univariate and multivariate GWAS analyses. Additionally, we investigated the functions of annotated genes associated with these traits. Key traits of interest included solidity of necrosis, convex hull area of root necrosis, ellipse eccentricity of root necrosis, percentage of necrosis, necrotic area fraction, and necrotic width fraction. Broad-sense and narrow-sense heritability estimates are key in providing insights into the genetic gain achievable through selection (Holland et al., 2002; Schmidt et al., 2019). Results showed that broad sense heritability estimates were low to moderate for all the root necrosis images traits while narrow sense heritability estimates were consistently low and ranged from 0.03 to 0.22. The low to moderate broad-sense heritability estimates provide evidence supporting the involvement of diverse levels of genetic factors in the manifestation of these root necrosis mage traits while the narrow sense heritability estimates also provide additional evidence supporting the involvement of diverse levels of additive genetic factors. These heritability estimates indicate some genetic influence, especially for root necrosis traits with moderate estimates. We suggest that random factors may affect the spatial distribution of necrotic lesions, leading to lower heritability estimates. Additionally, we excluded images without root necrosis from our analysis, but these images were given a score of one on a 1-5 scale. This could mean that the set of root necrosis images may not fully represent all the phenotypes, potentially resulting in underestimated heritability for certain image traits compared to the true genetic variation. Broad sense and narrow sense heritability estimates for root necrosis severity scores were similar with those previously reported (Okul Valentor et al., 2018; Ozimati et al., 2019, 2021; Nandudu et al., 2023).

Phenotypic and genetic correlations were analyzed to understand the genetic basis of root necrosis image traits and CBSD severities. We observed varied phenotypic and genetic correlations among root necrosis image traits, including significant positive correlations. Traits such as percentage of necrosis, necrotic width fraction, and necrotic area fraction, exhibited both high phenotypic and genotypic correlations (**Figure 4**). This means that these traits can be effective mutual predictors for indirect selection when quantifying root necrosis, resulting in cost efficiency, and reduced computational time. Nonetheless, for breeders to effectively utilize highly correlated root necrosis traits in breeding against root necrosis, it is essential to incorporate them into selection indexes (León et al., 2021). This would involve assigning appropriate weights to these traits based on their economic significance and heritability. Such indexes would enable breeders to prioritize and concentrate their efforts on the root necrosis traits that have the most substantial influence on overall crop enhancement. The high positive correlations between root necrosis image traits also signify the existence of common genetic mechanisms (Vattikuti et al., 2012; Sodini et al., 2018; van Rheenen et al., 2019).Correlations between root necrosis traits can be affected by various factors and one influencing factor is pleiotropism and linkage disequilibrium (Walsh & Blows, 2009; van Rheenen et al., 2019) Pleiotropism occurs when a single gene has an impact on multiple traits, leading to correlations between these traits. Another factor is linkage disequilibrium, a phenomenon described by Walsh and Blows (2009). In linkage disequilibrium, certain genetic variants tend to be inherited together, creating dependencies between traits. Both pleiotropism and linkage disequilibrium contribute to the complex relationships observed among different traits, highlighting the intricate nature of genetic interactions and their effects on trait correlations.

**Figure 7:**
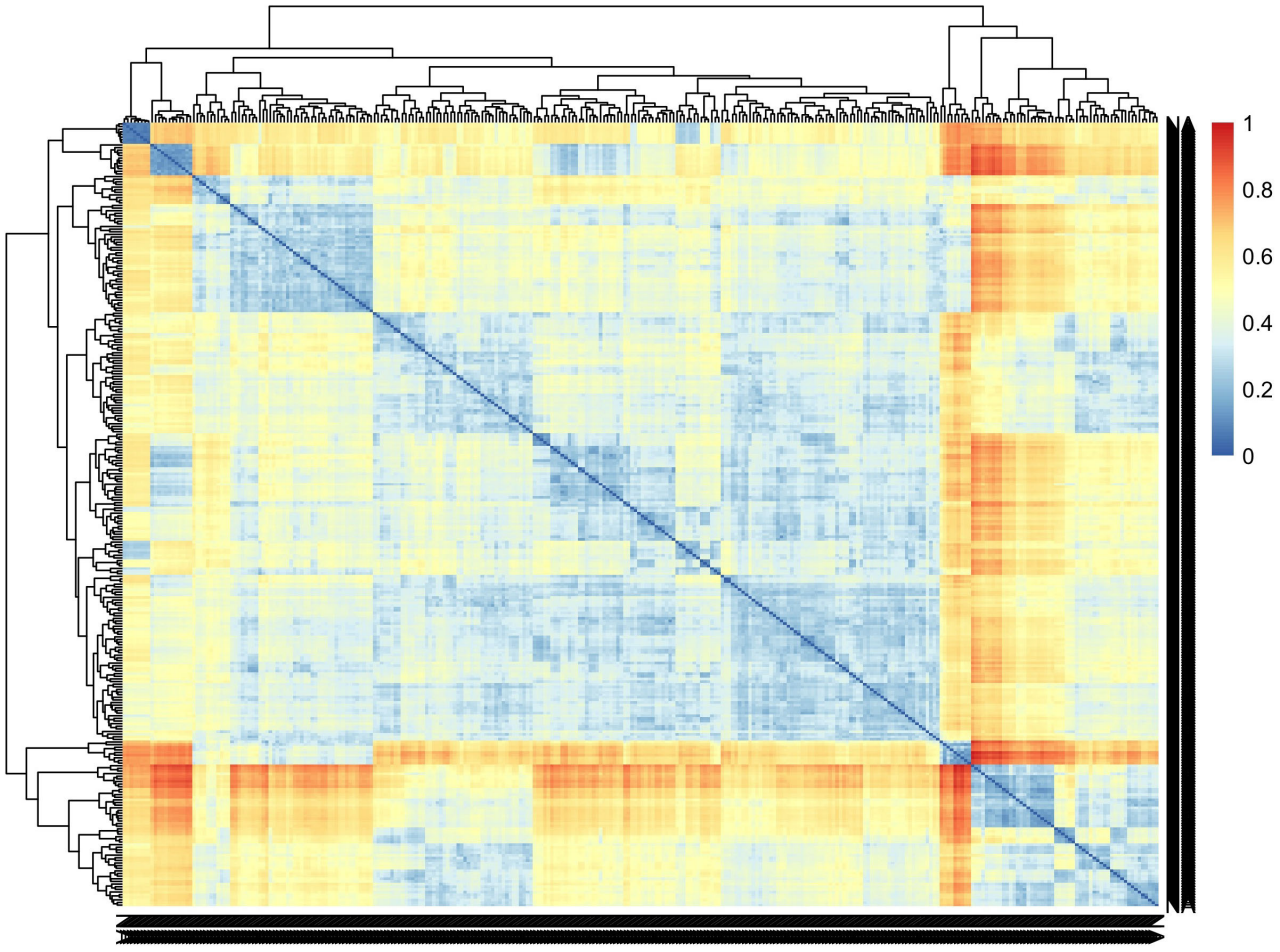
Pairwise genetic dissimilarities between clones in the C2 population were calculated using Euclidean distance. The lines were ordered using the average clustering method.

Incorporating genomic data into the analysis pipeline constitutes a pivotal strategy for unraveling the genetic foundations of CBSD root necrosis traits, even in scenarios characterized by low to moderate heritability estimates (Shao et al., 1991). We conducted a comprehensive analysis, encompassing both univariate and multivariate GWAS approaches. The univariate GWAS found eight SNP markers on chromosomes 1, 7, and 11, linked to both the ellipse eccentricity of root necrosis and the percentage of root necrosis. The haplotype view (**Supplementary Figure 11**) highlighted the 6 SNPs identified in chromosome 1 to be in a high LD block region (**Supplementary Figure 11A**). When we zoomed into the high LD block region where the 6 SNPs clustered, three of the SNPs were seen to be in high LD with correlations ranging from 0.9 to 0.97 (**Supplementary Figure 11B**). On average, four SNPs captured the variation explained by the significant SNPs identified on chromosome 1. Despite being significant, these SNPs only accounted for small proportions of phenotypic variance. These small proportions of variance could arise due to the complexity of CBSD root necrosis image traits’ genetic architecture which may arise from the influence of numerous rare variants with minor effects. Furthermore, standard GWAS methods may overlook low-frequency polymorphisms, which could also contribute to CBSD root necrosis image traits. Consequently, the significant SNPs identified in this study might only explain small proportions of phenotypic variance. The limited extent of phenotypic variability also suggests that the underlying causal variants linked to both root necrosis ellipse eccentricity and the percentage of root necrosis, despite their rarity, might reside at substantial distances from the significant SNPs pinpointed in this GWAS (Orozco et al., 2010; Wray et al., 2011). Consequently, the actual effect size could potentially be much more substantial than what is suggested by the significant SNPs identified in this study. To comprehend the impact of multiple rare variants, increasing the population size with diverse individuals is pivotal in enhancing statistical power for their detection. A larger sample size with diverse individuals ensures a more comprehensive representation of rare alleles, facilitating the identification of associations. In our study, the C2 population exhibited moderate genetic diversity (**Figure 7**). However, our sample size was significantly reduced by excluding root images lacking necrosis, potentially limiting our ability to detect multiple rare variants. Also, to determine causal SNPs linked to CBSD root necrosis, it is advised to establish a sizable biparental cassava population with diverse root necrosis levels for precise genetic mapping (Nzuki et al., 2017; Ferguson et al., 2019). This population should be evaluated in various environments to enhance statistical power. Fine mapping on regions of interest to identify causal variants associated with synthetic associations should be carried out. Also, integrating traditional QTL mapping with association mapping for a thorough understanding of the genetic architecture of CBSD root necrosis traits is key. We highlight that the outcomes of our study relating to root necrosis traits and CBSD severity scores align with earlier GWAS and QTL analyses focused on CBSD severities utilizing the 1-5 scoring method. These genomic regions include (1) chromosome 1 (Nandudu et al., 2023), (2) chromosome 11 (Kayondo et al., 2018), (3) chromosome 11 (Nzuki et al., 2017), chromosome 11 (Masumba et al., 2017), and chromosome 11 (Kawuki et al., 2016). This implies that certain genes or closely linked genes on these chromosomes might play a role in contributing root necrosis image traits and CBSD severity. Notably, such genes may also originate from prominent founders like Namikonga and Kiroba, which have been extensively employed in CBSD resistance breeding across multiple cassava breeding programs. Ultimately, the persistence of these genomic regions across various studies further emphasizes their significance for future research endeavors aimed at comprehending and enhancing resistance against cassava brown streak disease.

A total of 24 potential candidate genes were identified in the 172.2kb region on chromosome 1, with diverse range of functions crucial in the context of CBSD. These functions include ubiquitin-protein ligase activity, potentially involved in degrading viral components; ribosomal proteins, essential for viral replication and translation; transmembrane signal receptors, aiding in the recognition of viral molecules; DNA-binding transcription factors, governing the expression of defense-related genes; and RNA metabolism proteins, influencing viral RNA stability and processing. Molecular characterizations revealed various gene ontologies associated with these candidate genes. Catalytic activity (GO:0003824) indicates their roles in enzymatic reactions that can either facilitate or hinder CBSD viral replication and spread within cassava plants. The identification of transporter activity (GO:0005215) suggests that these genes may be involved in transporting CBSD viral molecules across cellular membranes, potentially increasing the severity of infection. Transcription regulator activity (GO:0140110) highlights the influence of these genes on gene expression regulation during CBSD viral responses. The biological classification of these potential candidate genes also highlighted their involvement in various critical processes critical for plant-virus interactions. These encompass cellular processes (GO:0009987), which are fundamental for viral entry and replication within the host. Metabolic processes (GO:0008152) underscore their roles in managing energy allocation and resources during infection, while biological regulation (GO:0065007) fine-tunes the plant’s responses to viral challenges. Processes related to localization (GO:0051179) are essential for precisely targeting viral components within the cell. Gene ontologies catalytic activity (GO:0003824), transporter activity (GO:0005215), structural molecule activity (GO:0005198) and molecular, cellular processes (GO:0009987), and metabolic processes (GO:0008152) were previously reported (Amuge et al., 2017) and their presence played a crucial role in facilitating susceptibility to CBSD. Therefore, our study sheds light on the complex nature of CBSD, its genetic architecture, and several candidate genes involved in root necrosis and disease severity. These findings have important implications for understanding CBSD and developing strategies for resistance breeding, which is critical for food security in East and Central Africa.

## Conclusions

Using PlantCV for root necrosis traits and a 1-5 scoring method for foliar and root severities in CBSD, we aimed to identify genomic regions associated with CBSD root necrosis through univariate and multivariate GWAS analyses. Key traits included solidity, convex hull area, ellipse eccentricity, percentage of necrosis, necrotic area fraction, and necrotic width fraction. Broad-sense heritability was low to moderate, indicating diverse genetic factors, while narrow-sense heritability was consistently low.

Phenotypic and genetic correlations were analyzed, revealing significant positive correlations among root necrosis traits, especially percentage of necrosis, necrotic width fraction, and necrotic area fraction. Integration of genomic data identified 8 significant SNPs associated with ellipse eccentricity and percentage of root necrosis, suggesting potential epistatic interactions with rare variants. A total of 24 candidate genes were found, involved in crucial processes like viral degradation, replication, transcription regulation, and defense-related gene expression. Gene ontologies highlighted their roles in catalytic activity, transporter activity, structural molecule activity, cellular and metabolic processes. These genes also played roles in cellular entry, energy allocation, regulatory responses, localization, homeostasis, reproduction, developmental and multicellular organismal processes, detoxification, and growth. Some of these ontologies were previously linked to CBSD susceptibility.

In conclusion our study has uncovered potential candidate genes linked to CBSD, shedding light on its genetic makeup and the intricacies of the disease. Despite our initial anticipation of enhanced heritability estimates and strong genomic associations through image analysis objectivity compared to the 1-5 scoring method, the results yielded unexpectedly lower outcomes than those achieved with the 1-5 scoring method. These findings hold considerable importance for comprehending CBSD and devising resistance breeding strategies, ultimately contributing to food security in East and Central Africa.

## Data availability statement

The datasets presented in this study can be found in online repositories. The names of the repository/repositories and accession number(s) can be found in the article/**Supplementary Material**.

## Author contributions

LN: Conceptualization, Data curation, Formal Analysis, Investigation, Methodology, Supervision, Visualization, Writing – original draft, Writing – review & editing. CS: Data curation, Formal Analysis, Methodology, Software, Writing – original draft, Writing – review & editing. AO: Formal Analysis, Methodology, Visualization, Writing – original draft, Writing – review & editing. KR: Conceptualization, Funding acquisition, Investigation, Methodology, Project administration, Resources, Supervision, Writing – original draft, Writing – review & editing. JJ: Conceptualization, Formal Analysis, Funding acquisition, Methodology, Project administration, Resources, Supervision, Validation, Writing – original draft, Writing – review & editing.

## Supplementary material

Supplementary Figure 1 Schematic of root necrosis processing steps; **(A)** root necrosis image after processing the root disc characteristics; **(B)** segmentation of root necrosis using L channel from the CIELAB (L*a*b*) (lightness, green-magenta, blue-yellow) color space; **(C)** morphological image processing; **(D)** measure of root necrosis.

Supplementary Figure 2 Quantile-quantile plots of; **(A)** EEN; Ellipse eccentricity of root necrosis, and **(B)** NECRO; Percentage of necrosis.

Supplementary Figure 3 Manhattan plots of -Log_10_(P) showing chromosomal positions of SNP markers in univariate GWAS associated with **(A)** SD; Solidity of necrosis, **(B)** CHAN; Convex hull area of root necrosis, **(C)** NAF; Necrotic area fraction, and **(D)** NWF; Necrotic width fraction The red line represents the significant threshold -Log_10_ (P) value of 5.1 which was determined by using the effective number of independent tests on each chromosome to modify the Bonferroni correction method.

Supplementary Figure 4 Quantile-quantile plots of; **(A)** SD; Solidity of necrosis, **(B)** CHAN; Convex hull area of root necrosis, **(C)** NAF; Necrotic area fraction, and **(D)** NWF; Necrotic width fraction.

Supplementary Figure 5 Manhattan plots of -Log_10_(P) showing chromosomal positions of SNP markers in univariate GWAS associated with **(A)** CBSDs3; CBSD foliar severity at 3 MAP, **(B)** CBSDs6: CBSD foliar severity at 6 MAP and CBSDs12: CBSD root severity at 12 MAP. The red line represents the significant threshold -Log_10_ (P) value of 5.112404 which was determined by using the effective number of independent tests on each chromosome to modify the Bonferroni correction method.

Supplementary Figure 6 Quantile-quantile plots of **(A)** CBSDs3; CBSD foliar severity at 3 MAP, **(B)** CBSDs6: CBSD foliar severity at 6 MAP and CBSDs12: CBSD root severity at 12 MAP.

Supplementary Figure 7 Manhattan plots of -Log_10_(P) showing chromosomal positions of SNP markers in multivariate GWAS associated with **(A)** convex hull area of root necrosis, percentage of necrosis, necrotic area fraction and necrotic width fraction, **(B)** necrotic area fraction and necrotic width fraction, **(C)** ellipse eccentricity of root necrosis, percentage of necrosis, necrotic area fraction and necrotic width fraction, **(D)** percentage of necrosis, necrotic area fraction and necrotic width fraction, **(E)** percentage of necrosis, necrotic area fraction and CBSD severity at 12 months after planting (CBSDS12).The red line represents the significant threshold -Log_10_ (P) value of 5.112404 which was determined by using the effective number of independent tests on each chromosome to modify the Bonferroni correction method.

Supplementary Figure 8 Quantile-quantile plots of **(A)** convex hull area of root necrosis, percentage of necrosis, necrotic area fraction and necrotic width fraction, **(B)** necrotic area fraction and necrotic width fraction, **(C)** ellipse eccentricity of root necrosis, percentage of necrosis, necrotic area fraction and necrotic width fraction, **(D)** percentage of necrosis, necrotic area fraction and necrotic width fraction, **(E)** percentage of necrosis, necrotic area fraction and CBSD severity at 12 months after planting (CBSDS12).

Supplementary Figure 9 Manhattan plots of -Log_10_(P) showing chromosomal positions of SNP markers in multivariate GWAS associated with **(A)** percentage of necrosis, CBSD foliar severity at 3 MAP, and CBSD foliar severity at 6 MAP and **(B)** percentage of necrosis and CBSD root severity at 12 MAP. The red line represents the significant threshold -Log_10_ (P) value of 5.112404 which was determined by using the effective number of independent tests on each chromosome to modify the Bonferroni correction method.

Supplementary Figure 10 Quantile-quantile plots of **(A)** percentage of necrosis, CBSD foliar severity at 3 MAP, and CBSD foliar severity at 6 MAP and **(B)** percentage of necrosis and CBSD root severity at 12 MAP.

Supplementary Figure 11
**(A)** Haplotype view chromosome 1 where the six SNPs associated to the percentage of necrosis were identified. (Red color intensity indicates the intensity of r^2^, i.e., higher color intensity means higher r^2^). **(B)** Haplotype view of the 172.2kb region on the chromosome 1 where the six SNPs associated to the percentage of necrosis were identified. (Red color intensity indicates the intensity of r^2^, i.e., higher color intensity means higher r^2^).
