# Supplementary figures and images for: Genetic analysis of cassava brown streak disease root necrosis using image analysis and genome-wide association studies
Source: Front Plant Sci. 2024 Mar 18;15:1360729. doi: 10.3389/fpls.2024.1360729 (PMC10982329; doi:10.3389/fpls.2024.1360729)

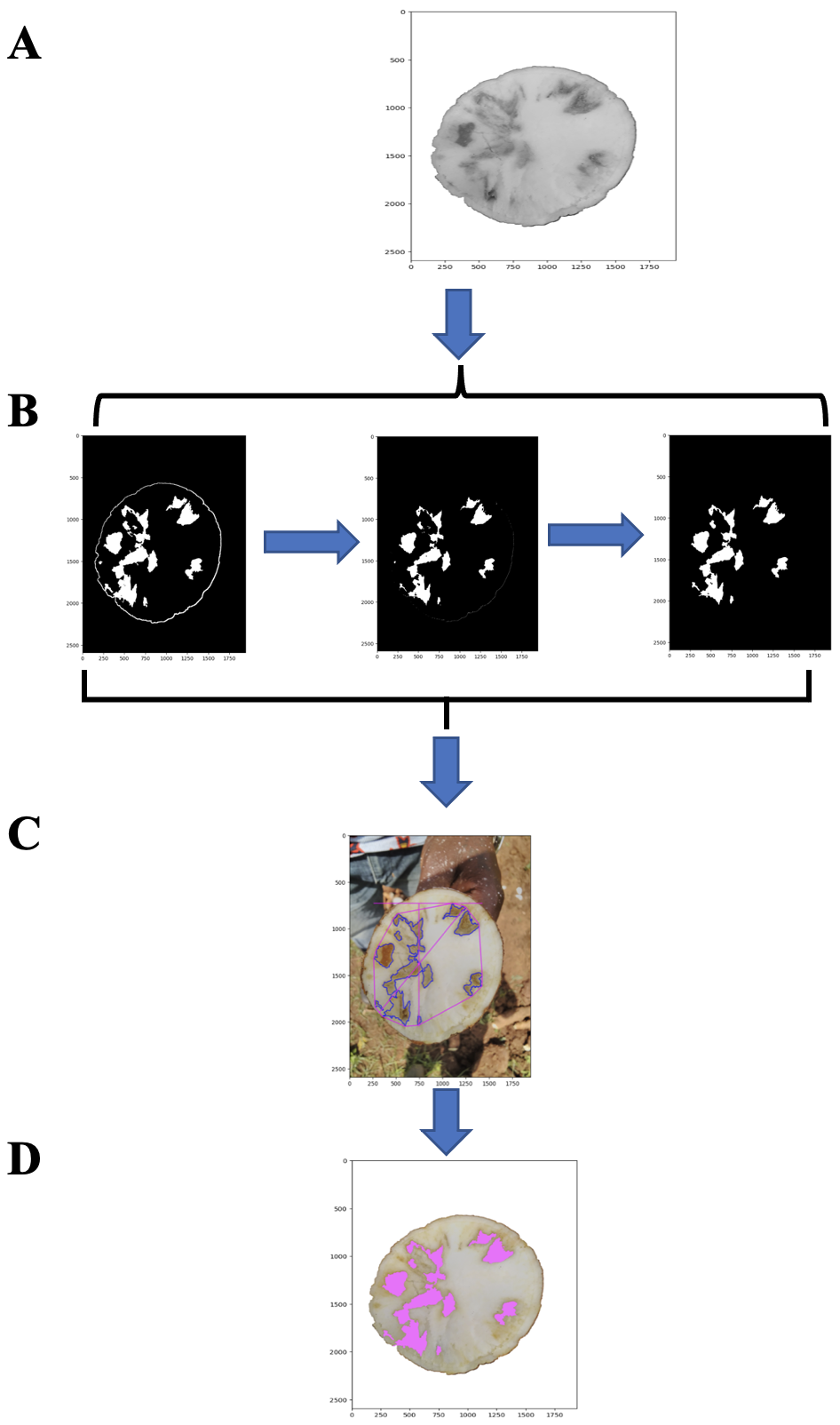

Supplement: Supplementary Figure 1 — Schematic of root necrosis processing steps; (A) root necrosis image after processing the root disc characteristics; (B) segmentation of root necrosis using L channel from the CIELAB (L*a*b*) (lightness, green-magenta, blue-yellow) color space; (C) morphological image processing; (D) measure of root necrosis. [file Image_1.png]

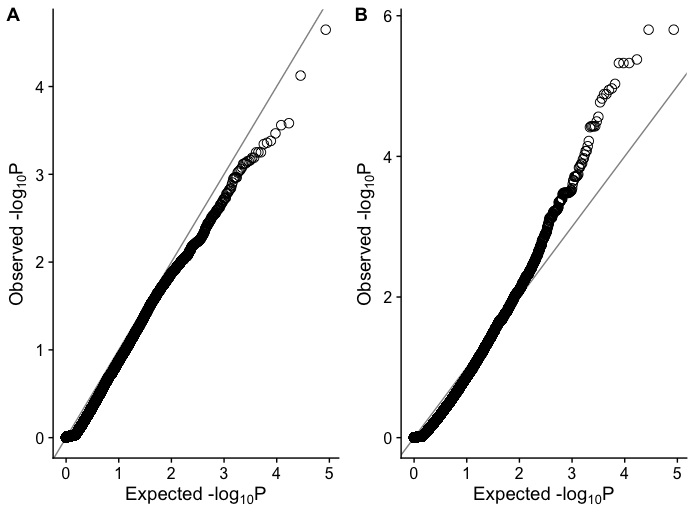

Supplement: Supplementary Figure 2 — Quantile-quantile plots of; (A) EEN; Ellipse eccentricity of root necrosis, and (B) NECRO; Percentage of necrosis. [file Image_2.png]

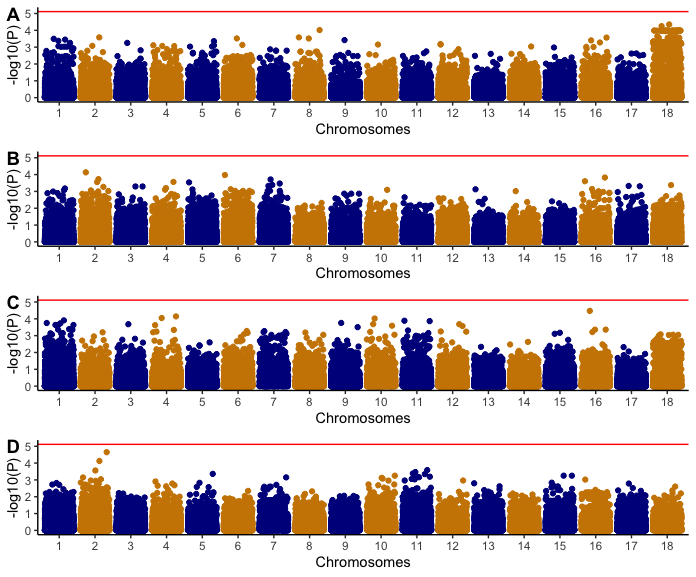

Supplement: Supplementary Figure 3 — Manhattan plots of -Log10(P) showing chromosomal positions of SNP markers in univariate GWAS associated with (A) SD; Solidity of necrosis, (B) CHAN; Convex hull area of root necrosis, (C) NAF; Necrotic area fraction, and (D) NWF; Necrotic width fraction The red line represents the significant threshold -Log10 (P) value of 5.1 which was determined by using the effective number of independent tests on each chromosome to modify the Bonferroni correction method. [file Image_3.png]

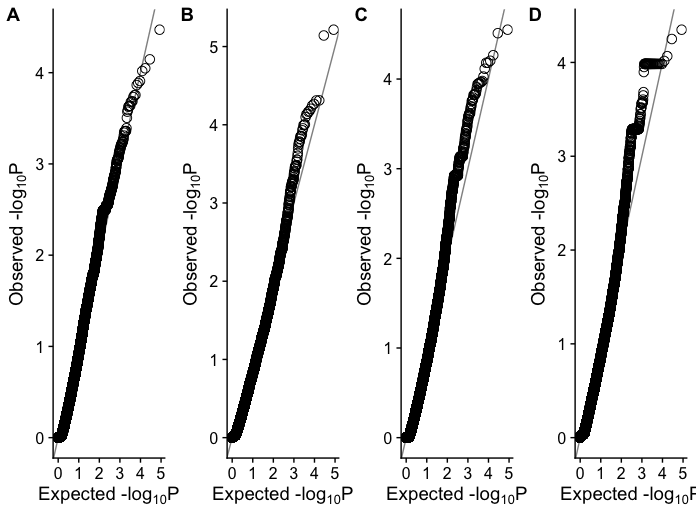

Supplement: Supplementary Figure 4 — Quantile-quantile plots of; (A) SD; Solidity of necrosis, (B) CHAN; Convex hull area of root necrosis, (C) NAF; Necrotic area fraction, and (D) NWF; Necrotic width fraction. [file Image_4.png]

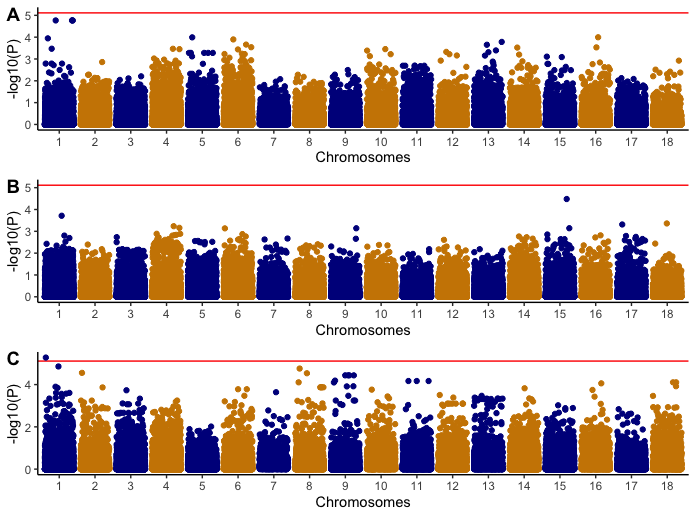

Supplement: Supplementary Figure 5 — Manhattan plots of -Log10(P) showing chromosomal positions of SNP markers in univariate GWAS associated with (A) CBSDs3; CBSD foliar severity at 3 MAP, (B) CBSDs6: CBSD foliar severity at 6 MAP and CBSDs12: CBSD root severity at 12 MAP. The red line represents the significant threshold -Log10 (P) value of 5.112404 which was determined by using the effective number of independent tests on each chromosome to modify the Bonferroni correction method. [file Image_5.png]

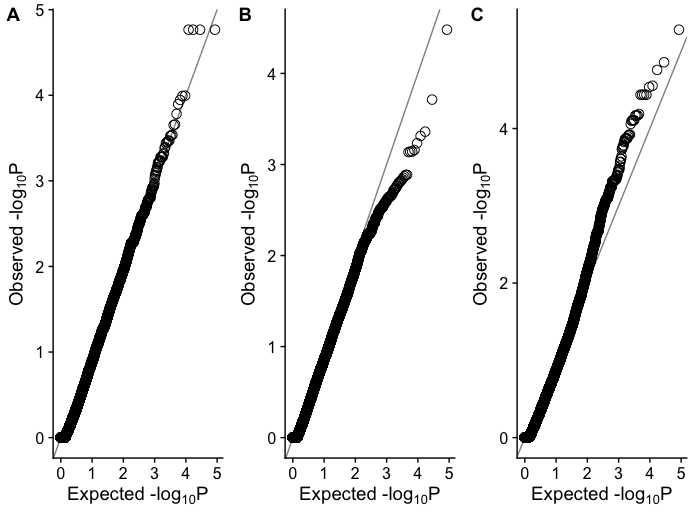

Supplement: Supplementary Figure 6 — Quantile-quantile plots of (A) CBSDs3; CBSD foliar severity at 3 MAP, (B) CBSDs6: CBSD foliar severity at 6 MAP and CBSDs12: CBSD root severity at 12 MAP. [file Image_6.png]

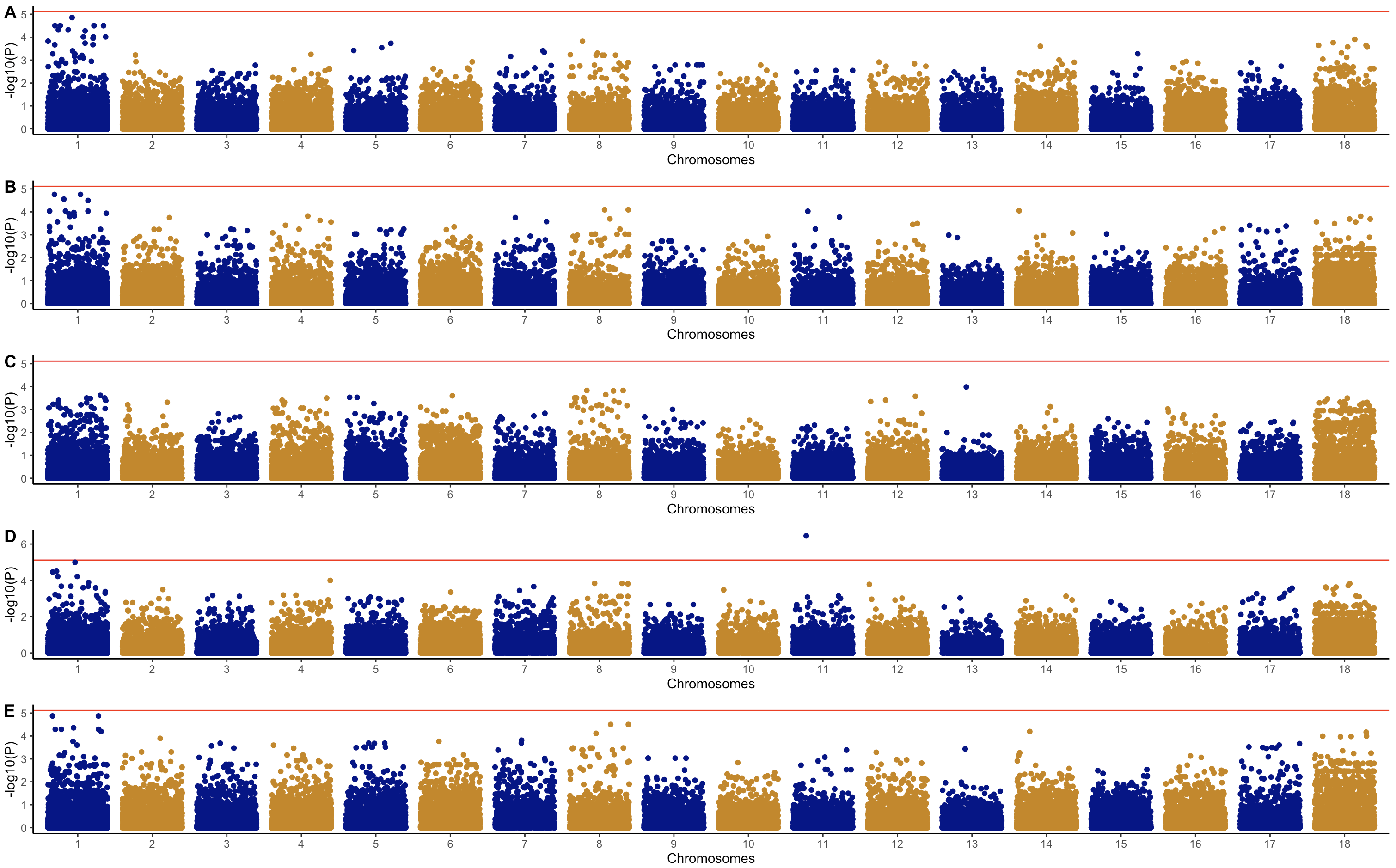

Supplement: Supplementary Figure 7 — Manhattan plots of -Log10(P) showing chromosomal positions of SNP markers in multivariate GWAS associated with (A) convex hull area of root necrosis, percentage of necrosis, necrotic area fraction and necrotic width fraction, (B) necrotic area fraction and necrotic width fraction, (C) ellipse eccentricity of root necrosis, percentage of necrosis, necrotic area fraction and necrotic width fraction, (D) percentage of necrosis, necrotic area fraction and necrotic width fraction, (E) percentage of necrosis, necrotic area fraction and CBSD severity at 12 months after planting (CBSDS12).The red line represents the significant threshold -Log10 (P) value of 5.112404 which was determined by using the effective number of independent tests on each chromosome to modify the Bonferroni correction method. [file Image_7.png]

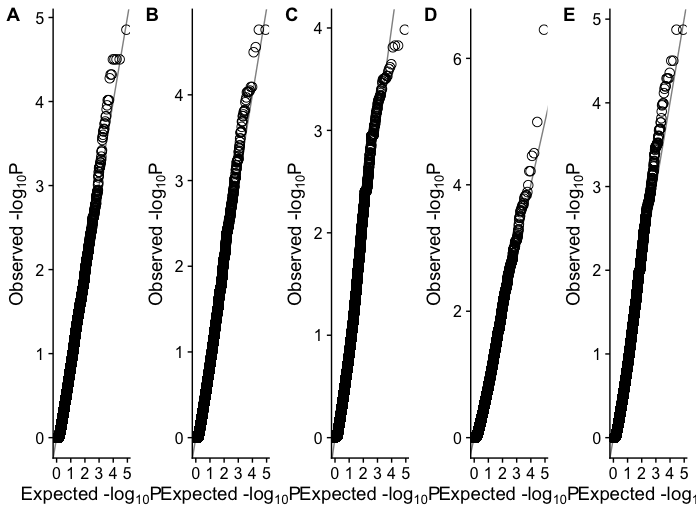

Supplement: Supplementary Figure 8 — Quantile-quantile plots of (A) convex hull area of root necrosis, percentage of necrosis, necrotic area fraction and necrotic width fraction, (B) necrotic area fraction and necrotic width fraction, (C) ellipse eccentricity of root necrosis, percentage of necrosis, necrotic area fraction and necrotic width fraction, (D) percentage of necrosis, necrotic area fraction and necrotic width fraction, (E) percentage of necrosis, necrotic area fraction and CBSD severity at 12 months after planting (CBSDS12). [file Image_8.png]

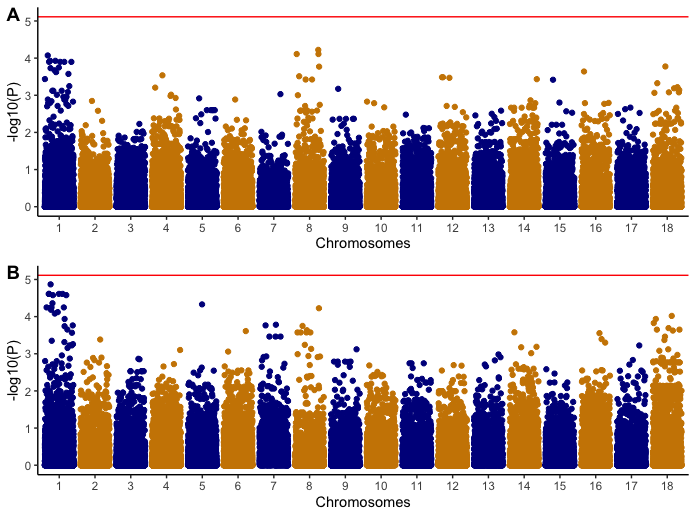

Supplement: Supplementary Figure 9 — Manhattan plots of -Log10(P) showing chromosomal positions of SNP markers in multivariate GWAS associated with (A) percentage of necrosis, CBSD foliar severity at 3 MAP, and CBSD foliar severity at 6 MAP and (B) percentage of necrosis and CBSD root severity at 12 MAP. The red line represents the significant threshold -Log10 (P) value of 5.112404 which was determined by using the effective number of independent tests on each chromosome to modify the Bonferroni correction method. [file Image_9.png]

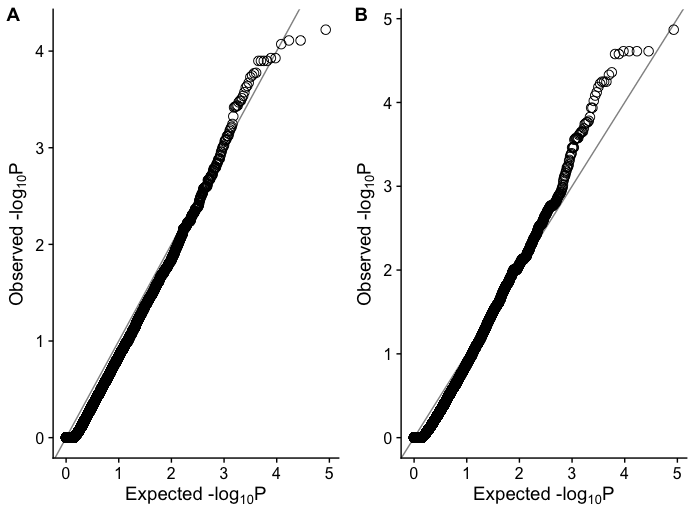

Supplement: Supplementary Figure 10 — Quantile-quantile plots of (A) percentage of necrosis, CBSD foliar severity at 3 MAP, and CBSD foliar severity at 6 MAP and (B) percentage of necrosis and CBSD root severity at 12 MAP. [file Image_10.png]

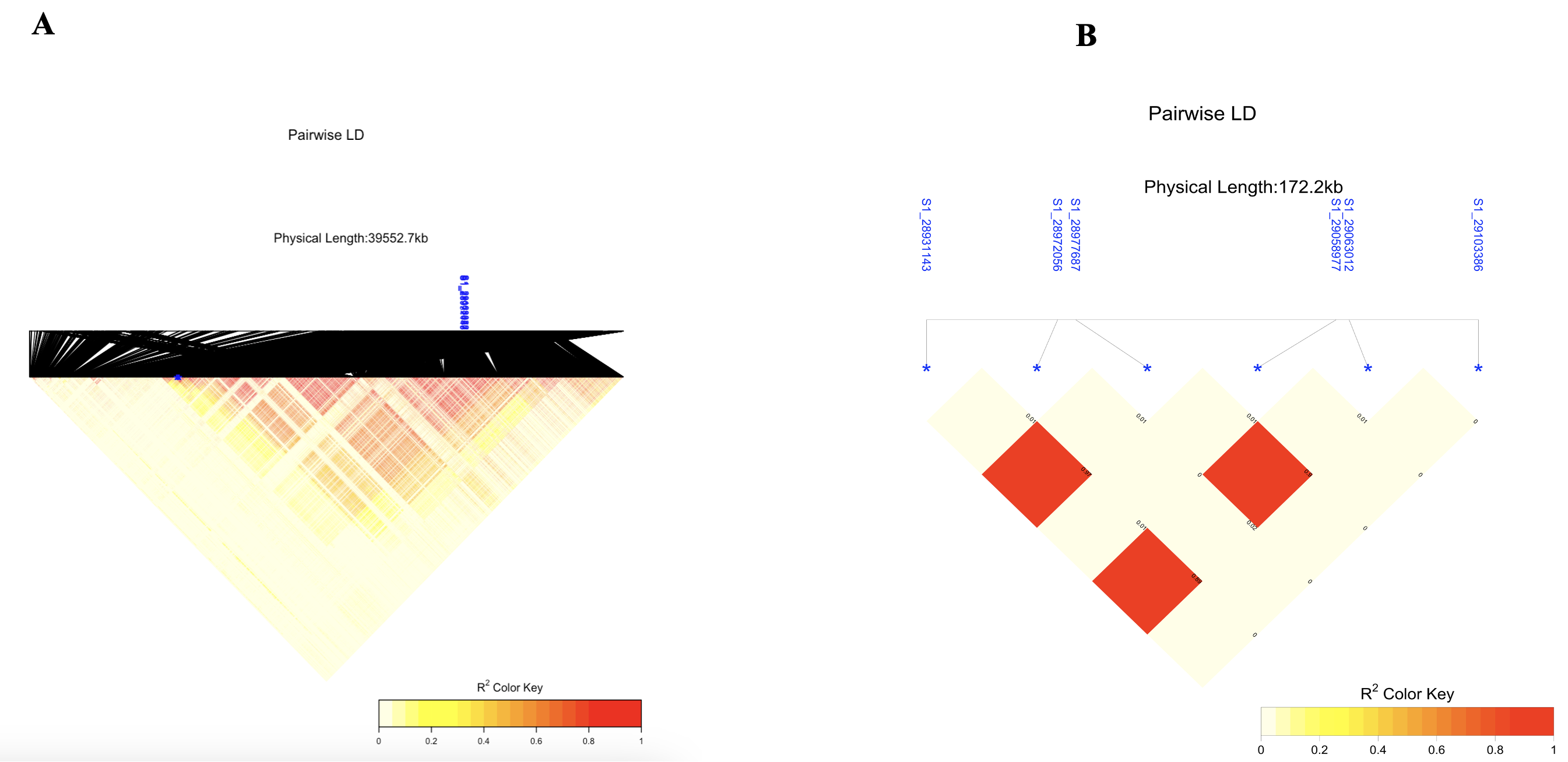

Supplement: Supplementary Figure 11 — (A) Haplotype view chromosome 1 where the six SNPs associated to the percentage of necrosis were identified. (Red color intensity indicates the intensity of r2, i.e., higher color intensity means higher r2). (B) Haplotype view of the 172.2kb region on the chromosome 1 where the six SNPs associated to the percentage of necrosis were identified. (Red color intensity indicates the intensity of r2, i.e., higher color intensity means higher r2). [file Image_11.png]
