# Supplementary material for: Genetic analysis of cassava brown streak disease root necrosis using image analysis and genome-wide association studies
Source: Front Plant Sci. 2024 Mar 18;15:1360729. doi: 10.3389/fpls.2024.1360729 (PMC10982329; doi:10.3389/fpls.2024.1360729)
Supplement: Supplementary file 13 [file DataSheet_1.pdf]

Cassava

August 12, 2022

[1]:
```
#Set the notebook display method
# inline = embedded plots, notebook = interactive plots
%matplotlib inline

#Import Statements- "matplotlib" is used for backend plotting in Jupyter␣
 ↪notebooks
from plantcv import plantcv as pcv
import matplotlib
```

[2]:
```
#Set figure size for the notebook
matplotlib.rcParams["figure.figsize"]= (8,8)
```

[5]:
```
#Set up code that will help this script to eventually run in Parallel

#The options class mimics the workflow command-line argument parser
#that is used for workflow parallelization. Using it while developing
#a workflow in Jupyter makes it easier to convert the workflow to a script later.
class options:
    def __init__(self):
        self.image = "./
 ↪C2_NaCRRI_CET_GWAS_2019_409_8_Root1_image2_1_2020-10-22-10-50-27.jpg" #put the␣
 ↪images in the same location as the jupyter notebook
        self.debug = "plot" #Set self.debug to "plot" so that image outputs for␣
 ↪each step is shown
        self.writeimg = False
        self.result = "cassava_results"
        self.outdir = "."

#get options
args = options()

#set debug to the global parameter
pcv.params.debug = args.debug

#increase text size in plots
pcv.params.text_size = 5
pcv.params.text_thickness = 20
```

[6]:
```
#Read the image
img1, path, filename = pcv.readimage(filename=args.image)
```

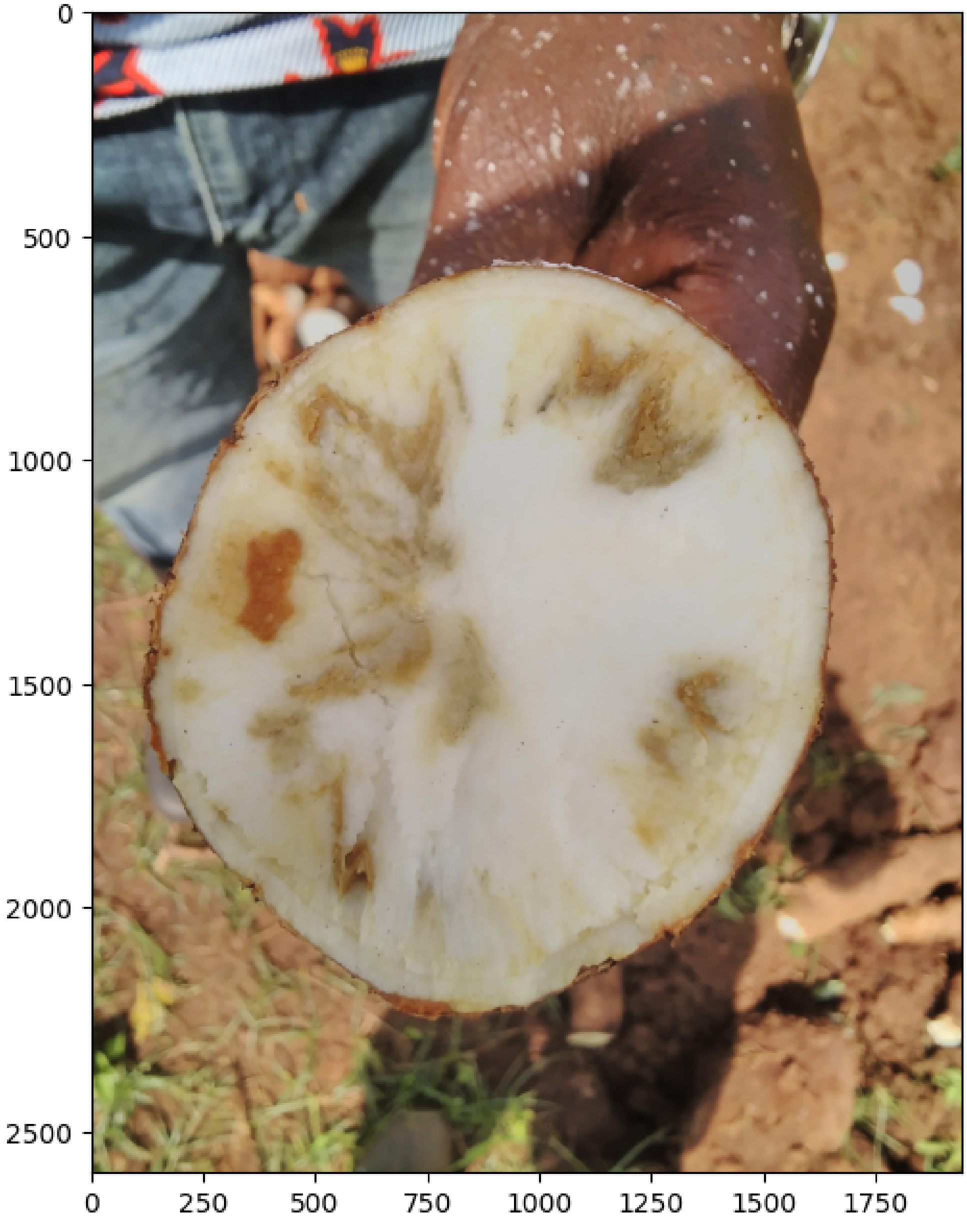

[7]:
```
#Step 3: segment object from the background
#First begin by looking at each of the channels from the HSV and LAB color spaces
#This will hlep us decide what colorspace/channels will help us best threshold␣
↪the object of interest
```

```
cs_plot = pcv.visualize.colorspaces(rgb_img=img1, original_img=True) #this is a␣
↪tool to show the image in different colorspaces to help us find what channel␣
↪will work best for thresholding
```

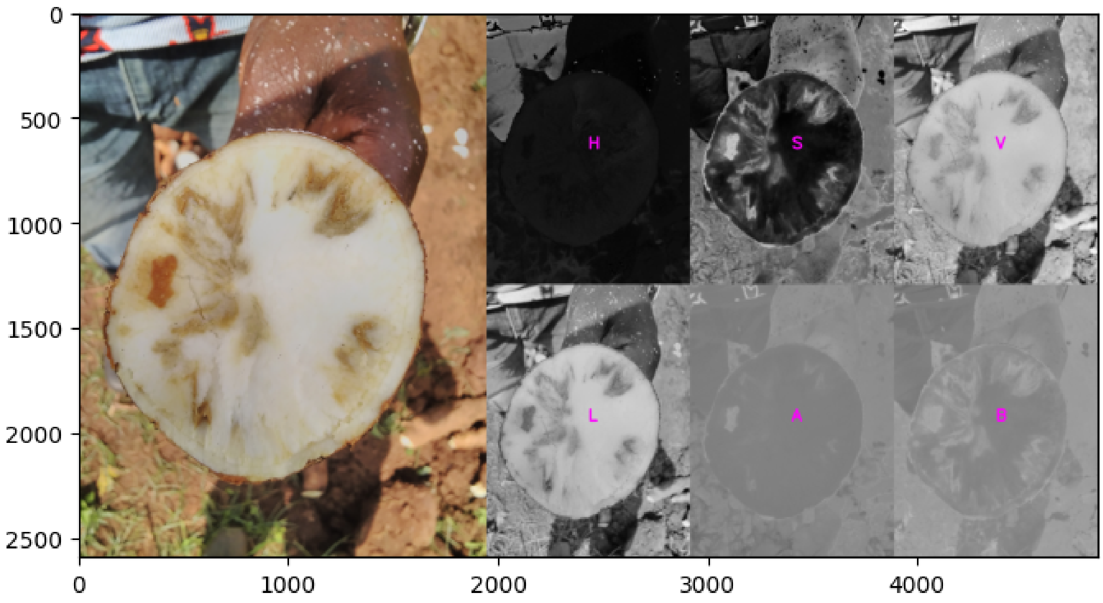

[8]:
```
#lets look closer at thresholding based on "l" from L*a*b* colorspace
l = pcv.rgb2gray_lab(rgb_img=img1, channel="l")
```

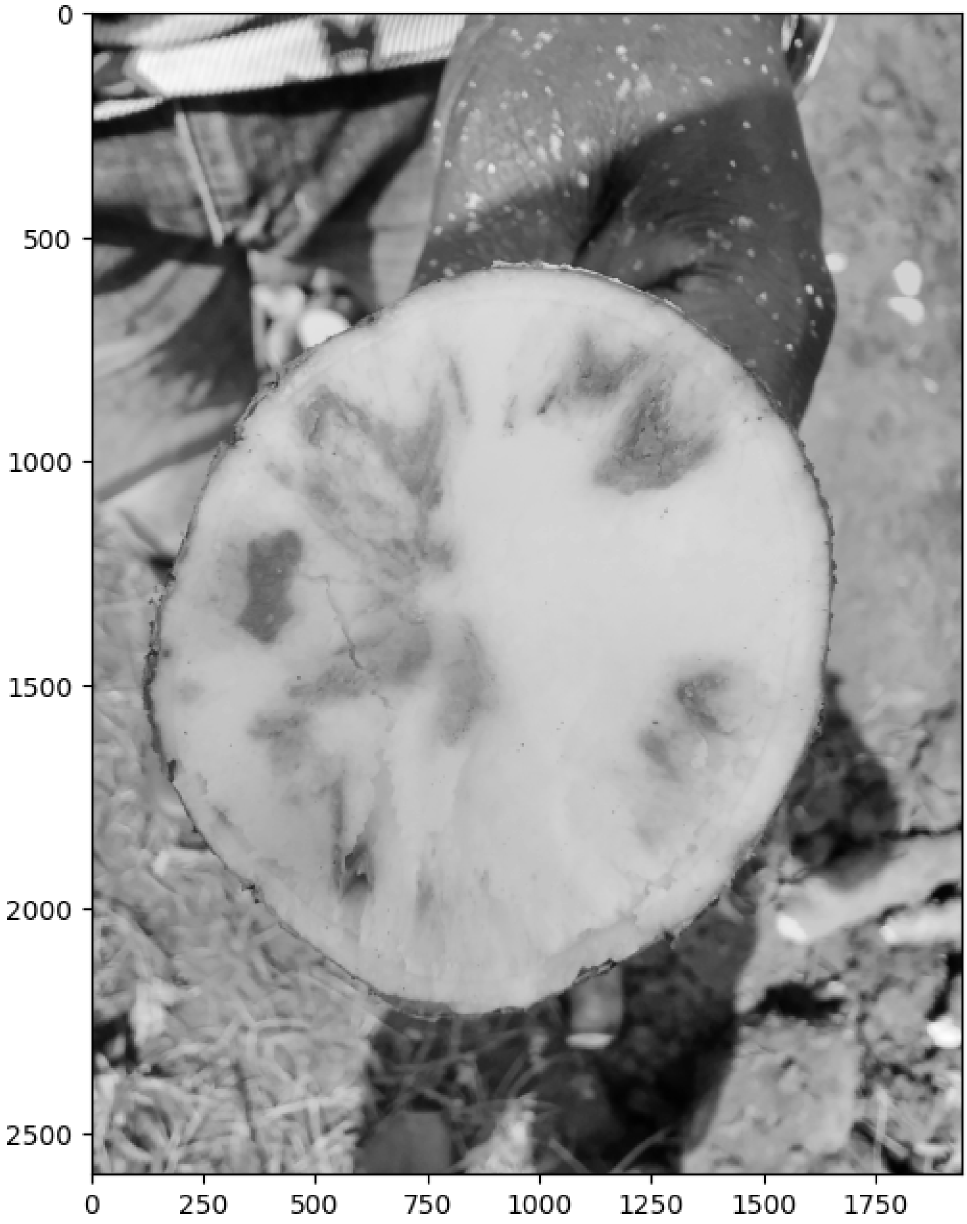

[9]:
```
#now threshold on the "l" channel
masked2, masked = pcv.threshold.custom_range(img=l, lower_thresh=[160],␣
↪upper_thresh=[255])
```

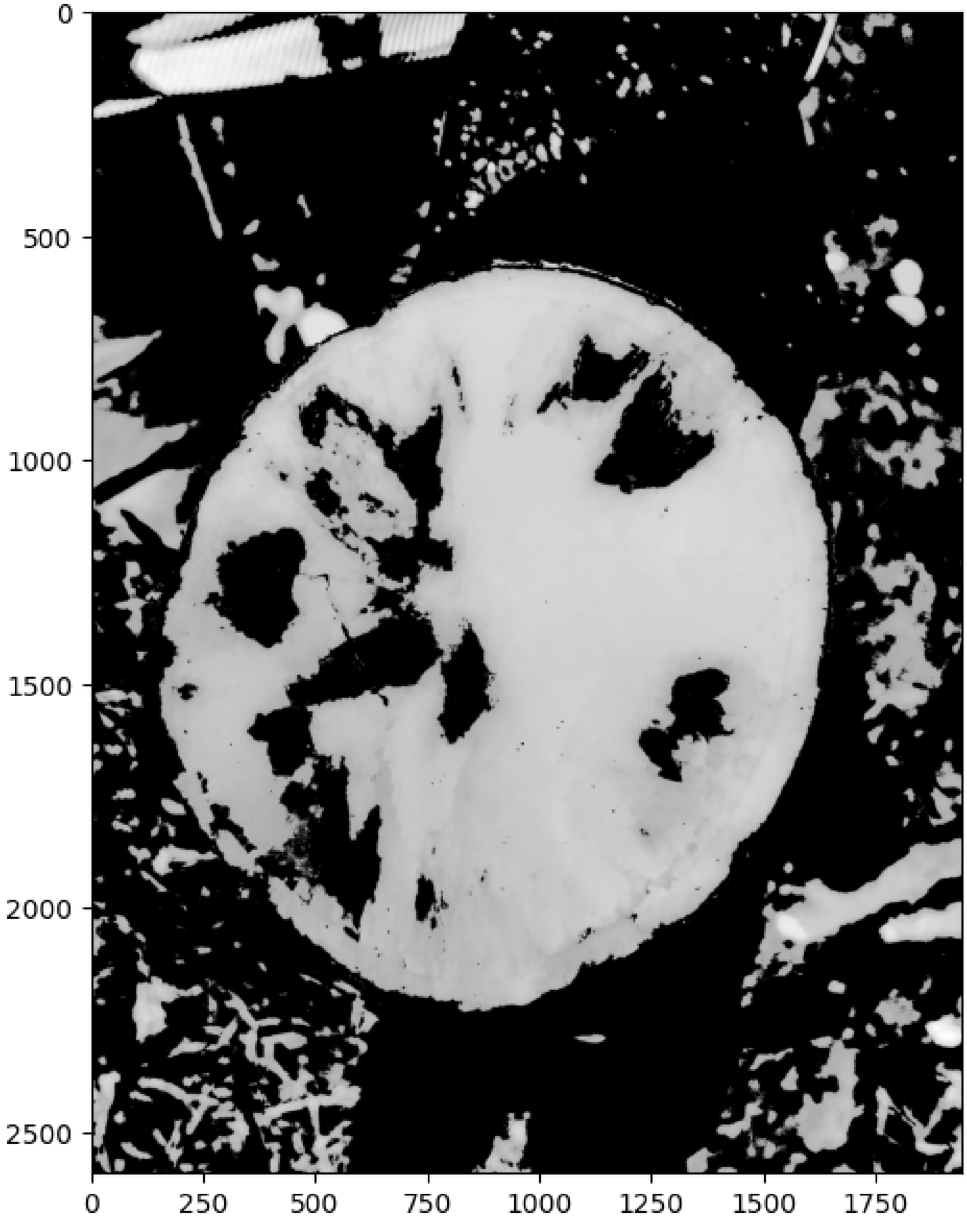

0
500
1000
1500
2000
2500
0
250
500
750
1000
1250
1500
1750

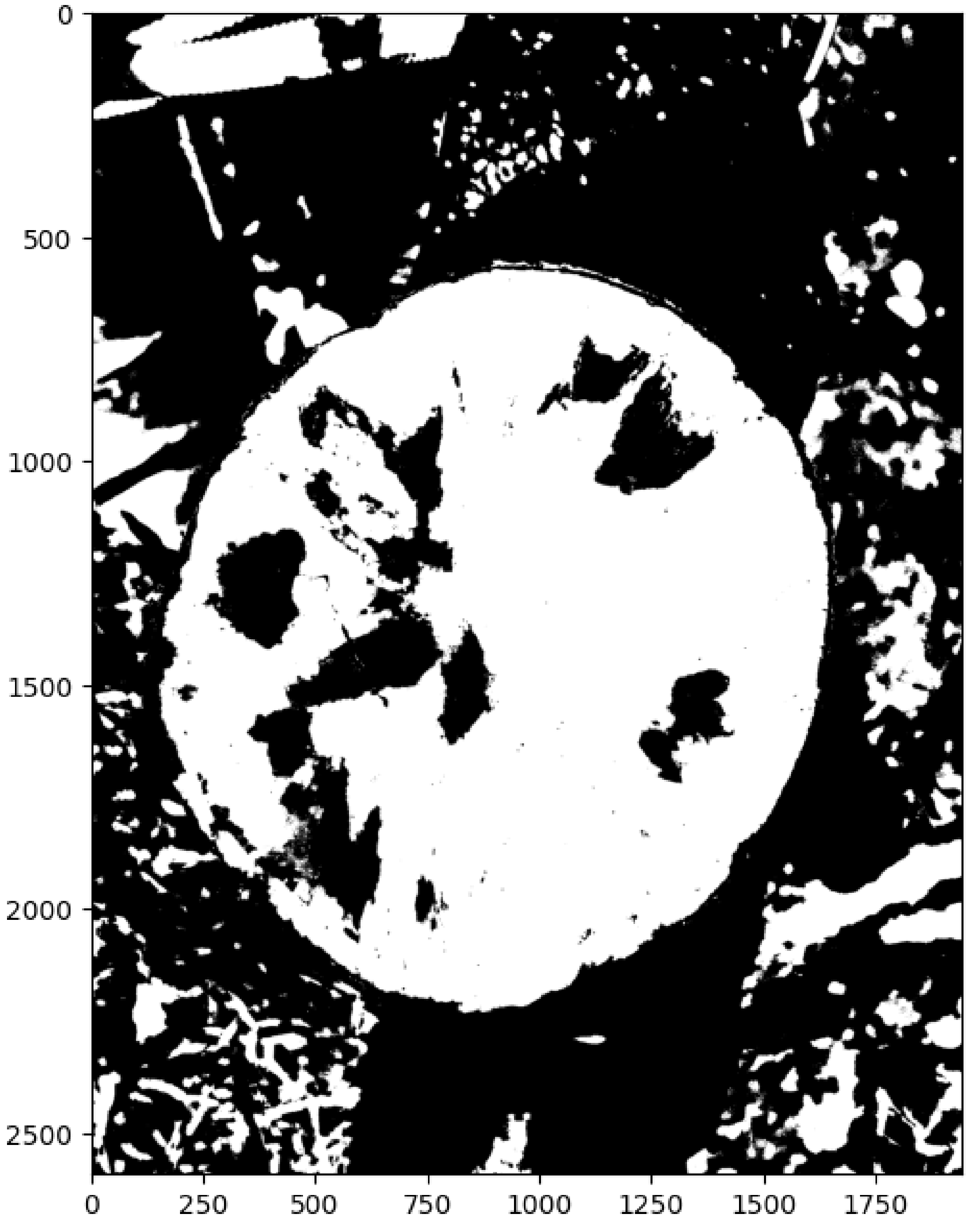

[10]:
```
# Fill in small objects/noise in the background of the image
binary_img = pcv.median_blur(gray_img=masked2, ksize=5)

filled = pcv.fill(bin_img=binary_img, size=100000)
```

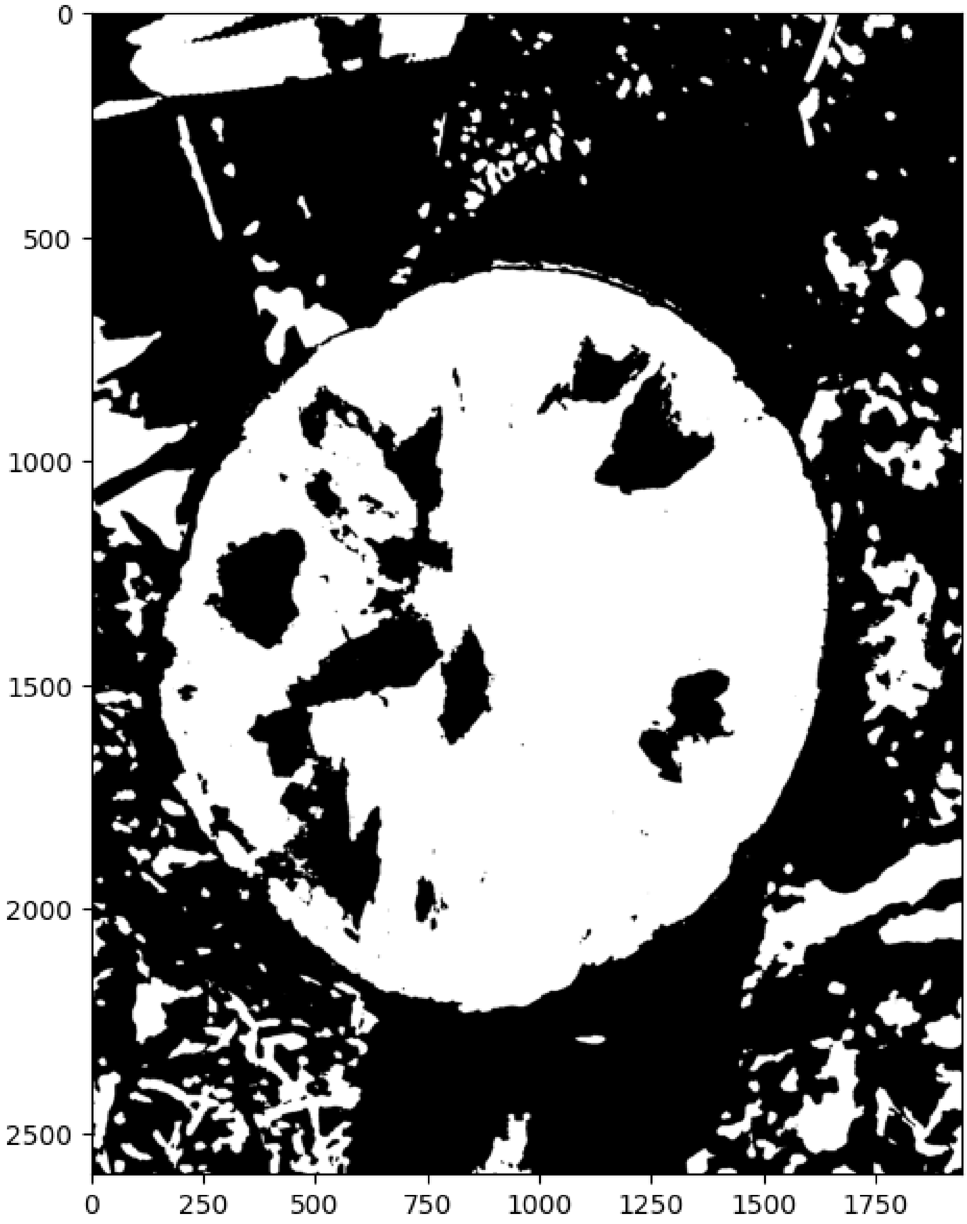

0
500
1000
1500
2000
2500
0
250
500
750
1000
1250
1500
1750

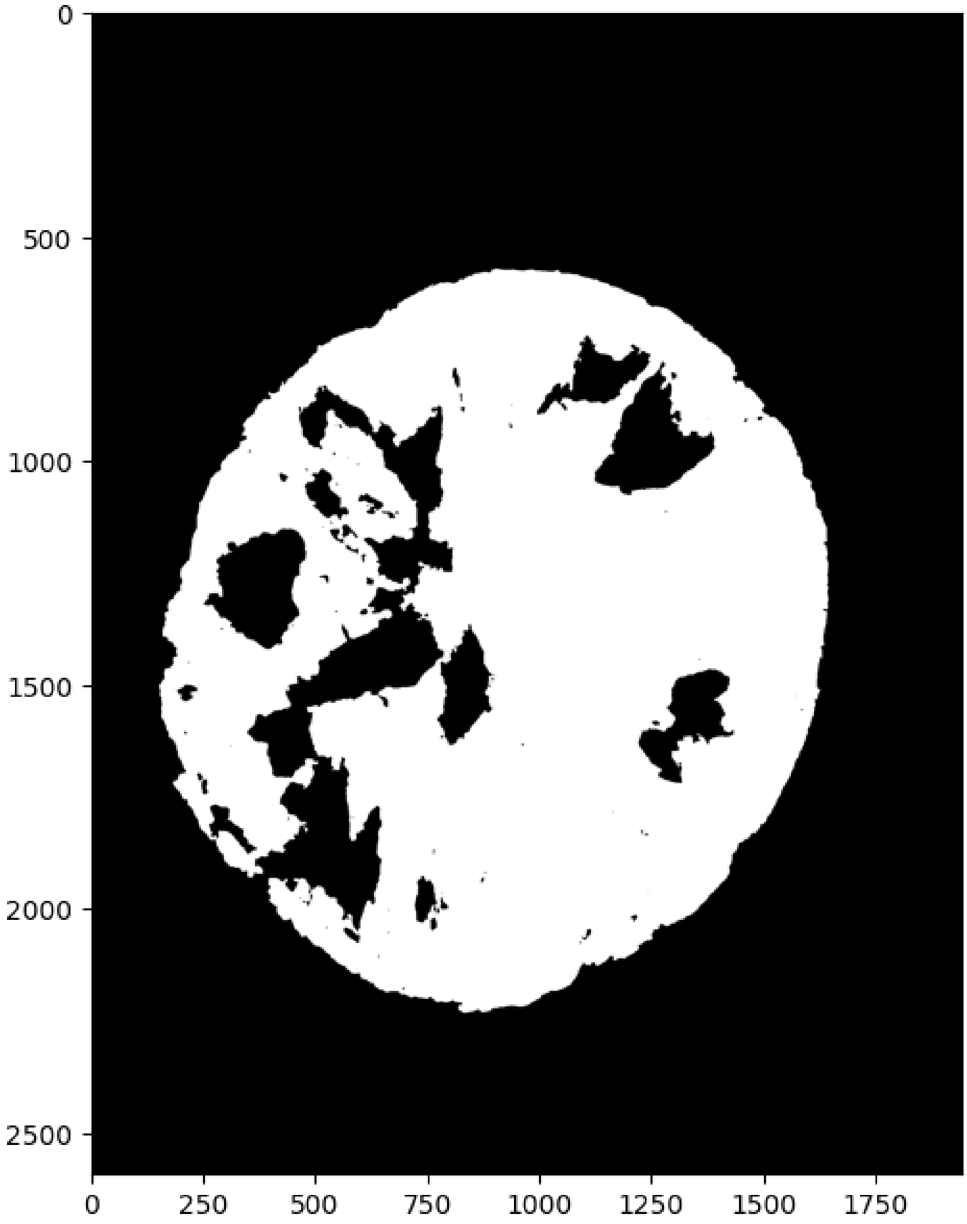


```
[11]: # Dilate to help reduce holes in object
      dilated_crosssection = pcv.dilate(gray_img=filled, ksize=4, i=5)
```

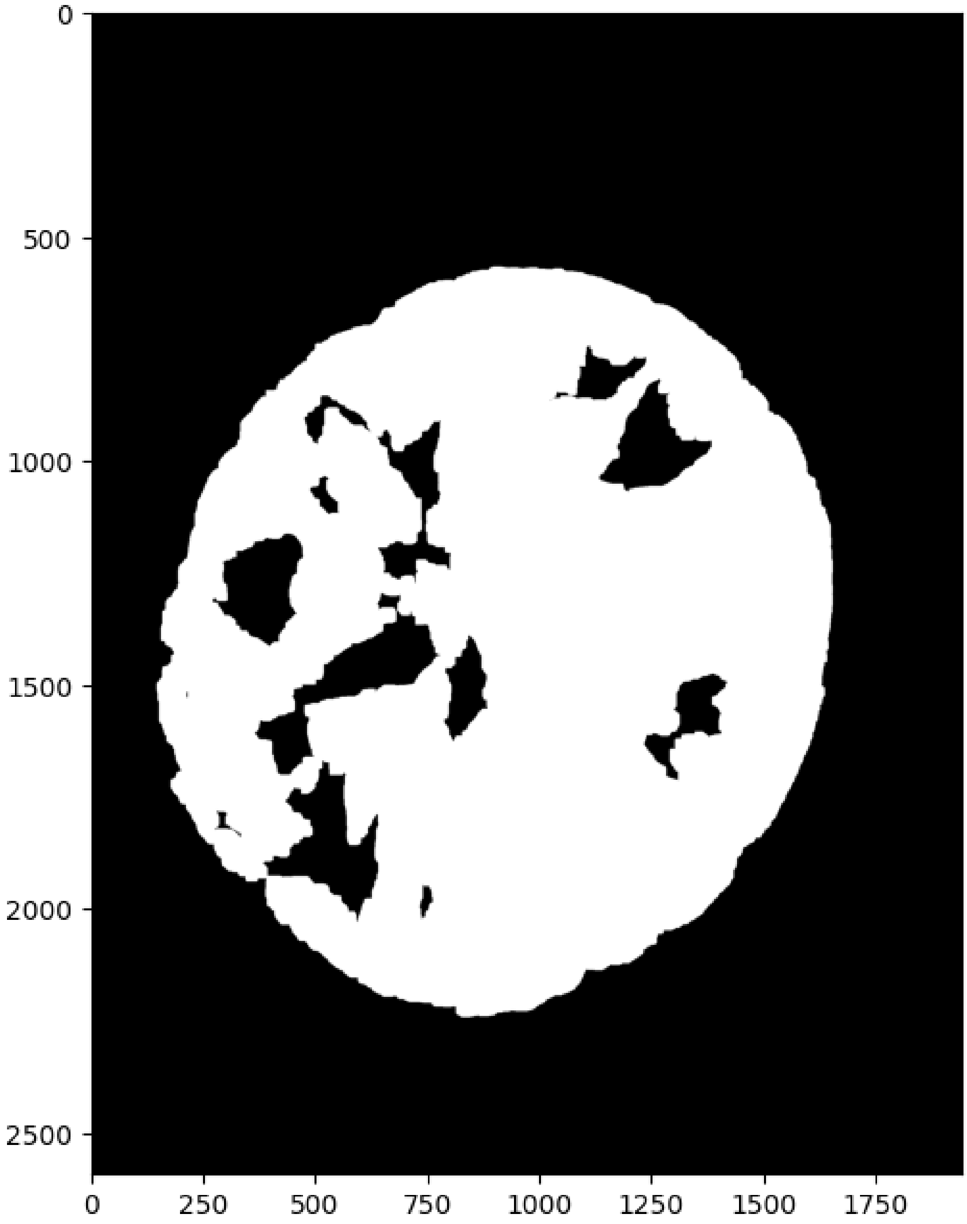

[12]:
```
#Fill holes to capture the entire cross section
Total_Cross_Section = pcv.fill_holes(bin_img=dilated_crosssection)
```

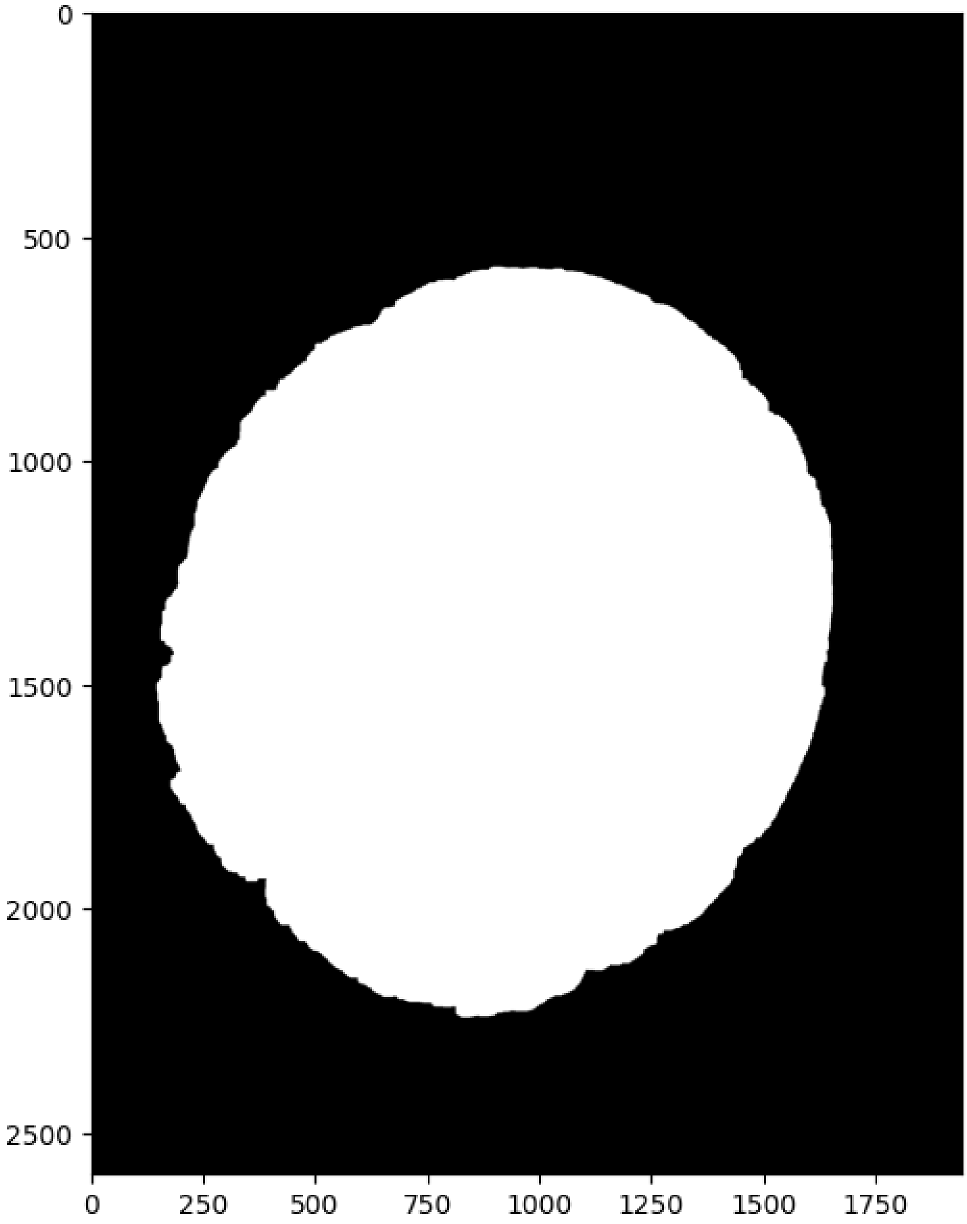

[13]:
```
# Apply mask to the original image to see what we capture as the␣
↪"total_cross_section"
masked1 = pcv.apply_mask(img=img1, mask=Total_Cross_Section, mask_color='white')
```

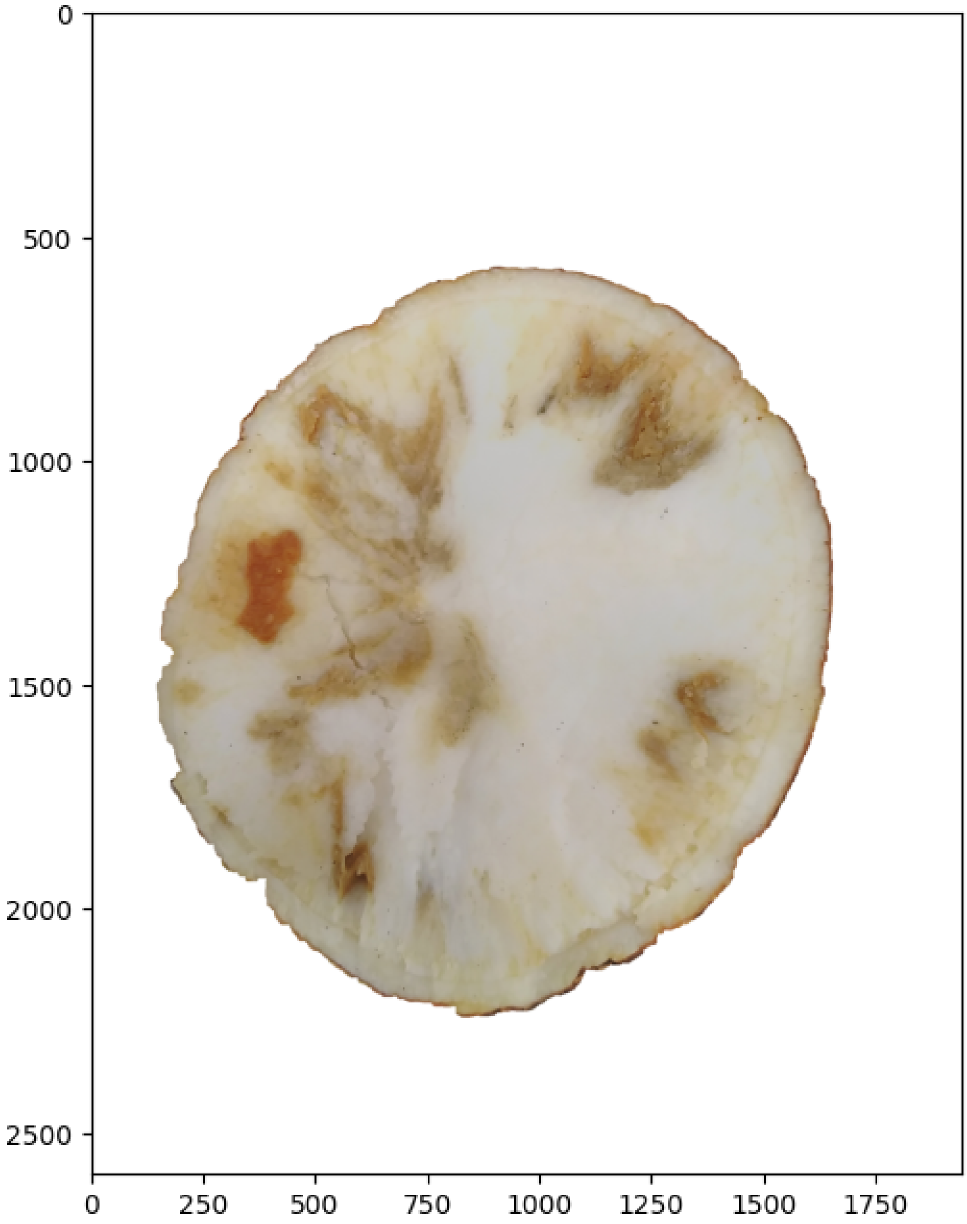

[14]:
```
#Find objects
objects1, hierarchy1 = pcv.find_objects(img=masked1, mask=Total_Cross_Section)
```

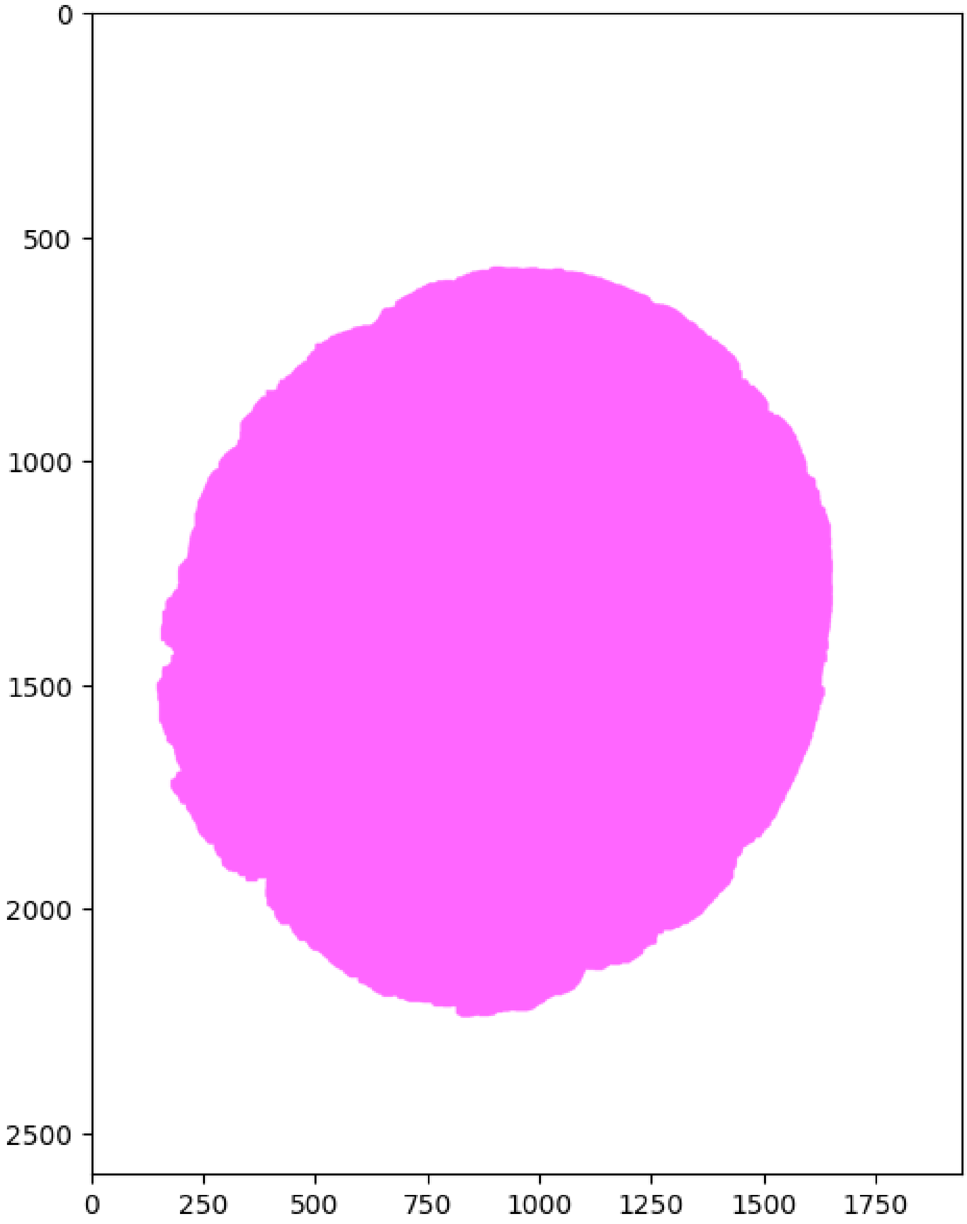

[15]:
```
#combine kept objects
obj1, mask1 = pcv.object_composition(img=img1, contours=objects1,␣
 ↪hierarchy=hierarchy1)
```

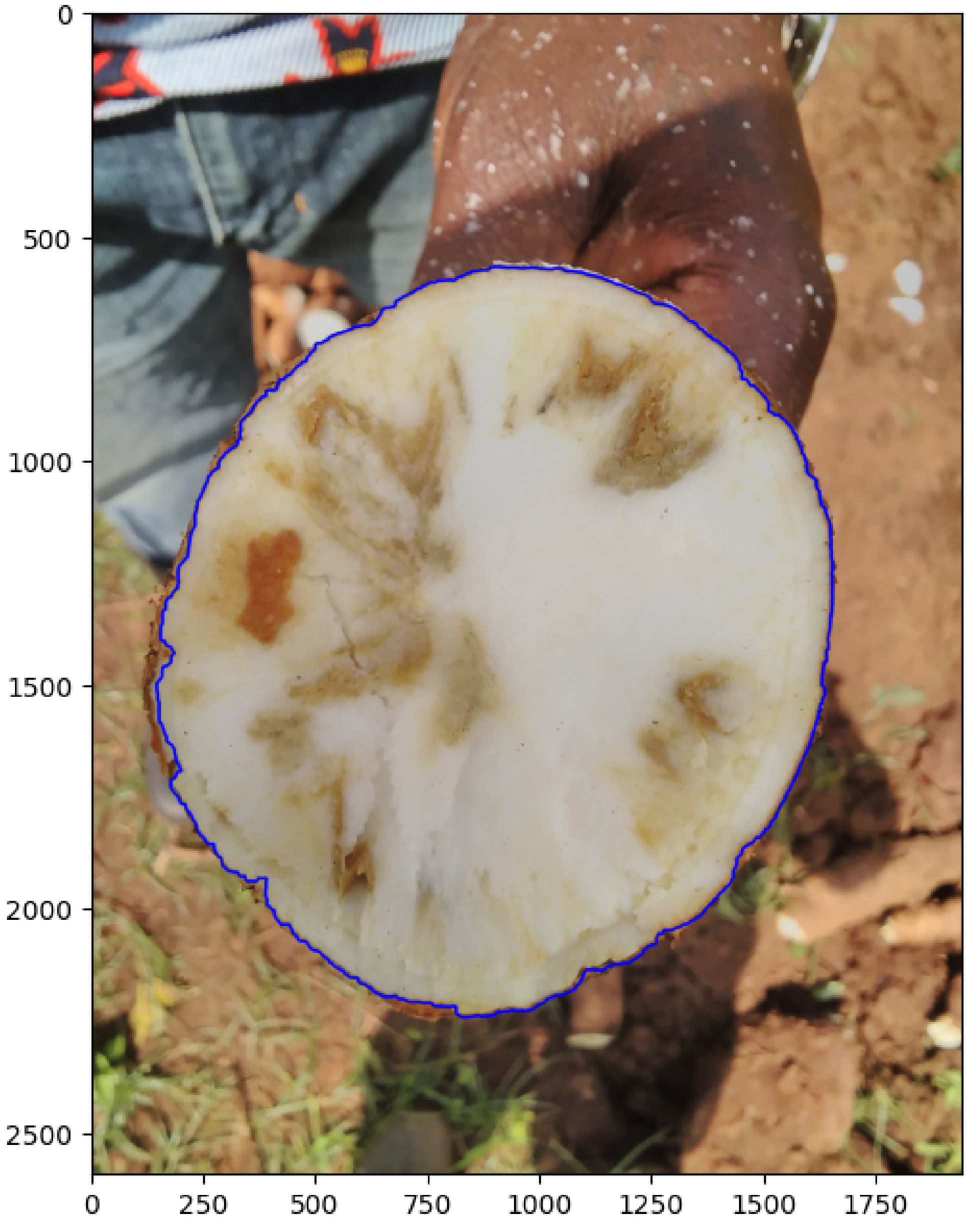
0
500
1000
1500
2000
2500
0
250
500
750
1000
1250
1500
1750

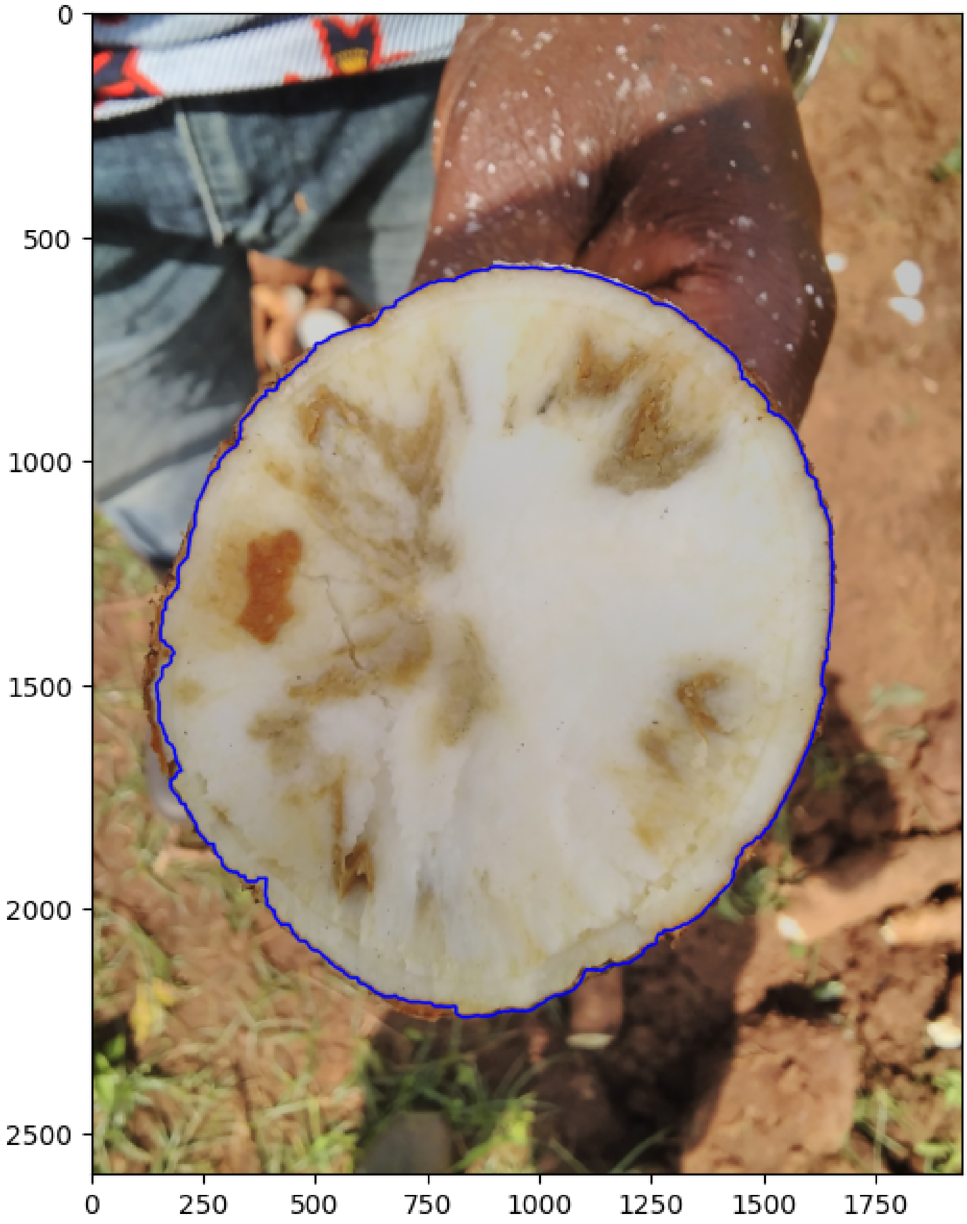

[16]:
```
############### Analysis ################
# Find shape properties of the cross section, data gets stored to an output␣
↪class automatically
analysis_image1 = pcv.analyze_object(img=img1, obj=obj1, mask=mask1,␣
↪label="total_cross_section")
```

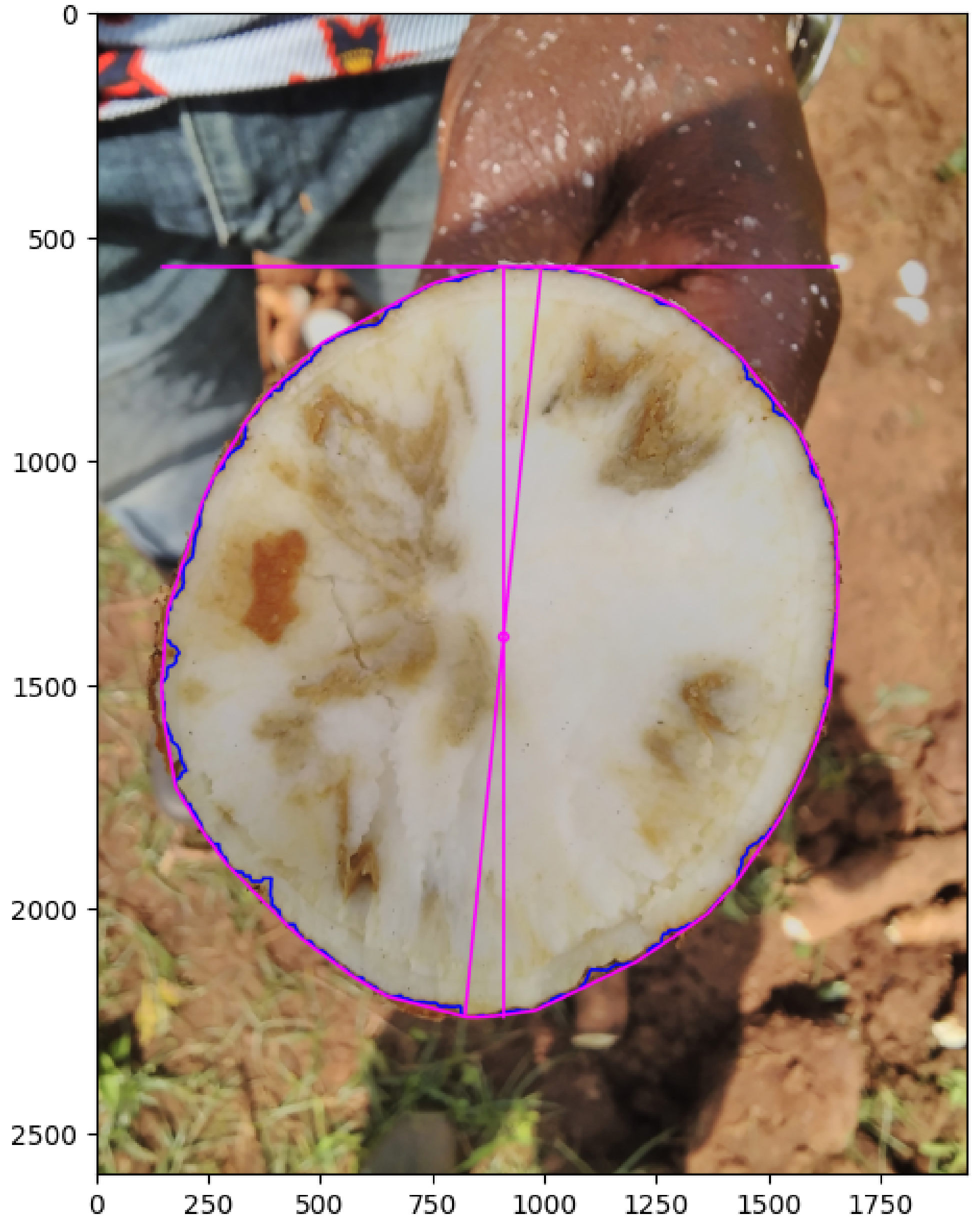


```
[17]: #lets look closer at thresholding based on "l" from L*a*b* colorspace to see if␣
      ↪we segment out necrosis
      l = pcv.rgb2gray_lab(rgb_img=masked1, channel="l")
```

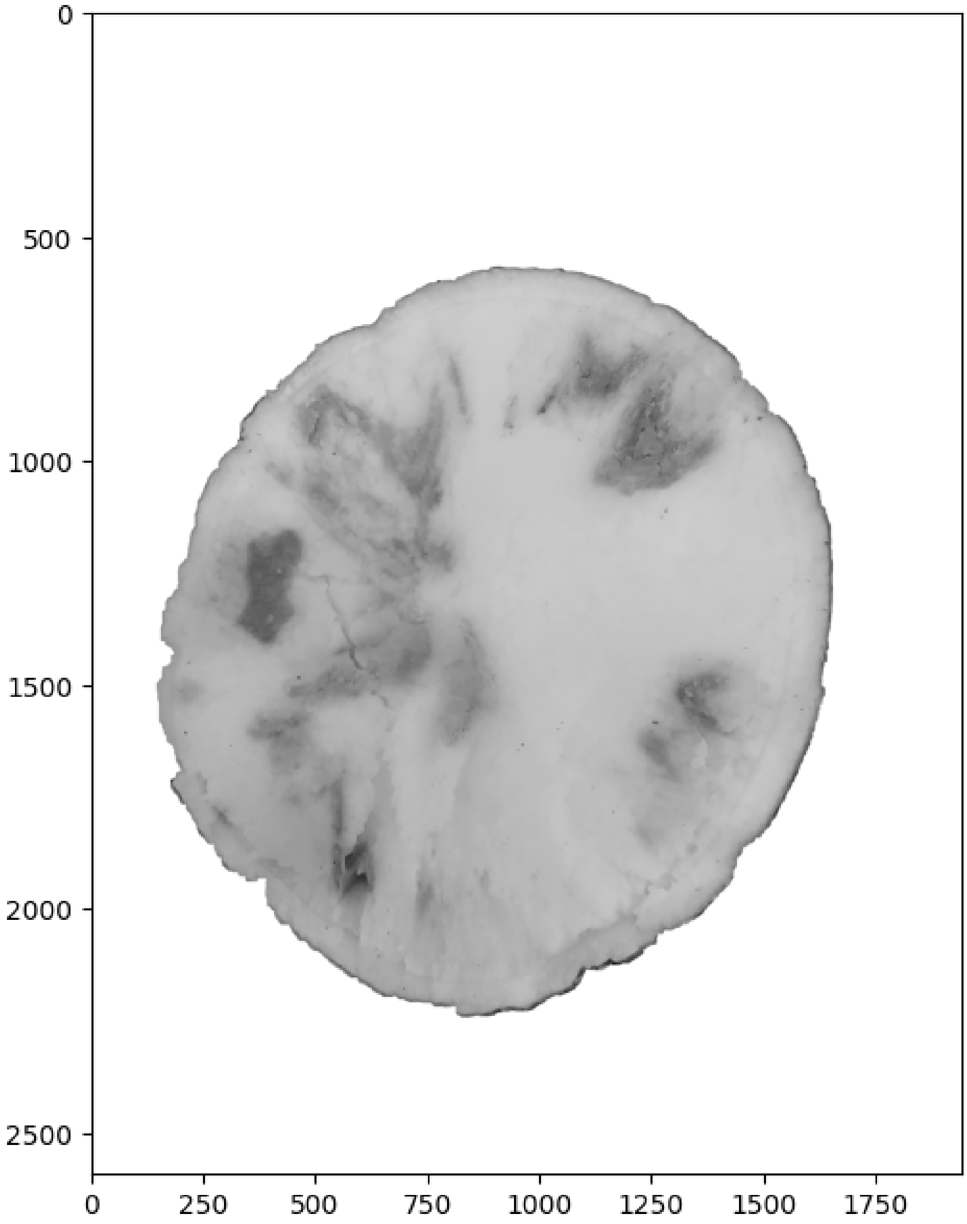

[18]:
```
#lets try to threshold out the necrosis by the "l" channel
mask3, masked3 = pcv.threshold.custom_range(img=l, lower_thresh=[20],␣
 ↪upper_thresh=[160])
```

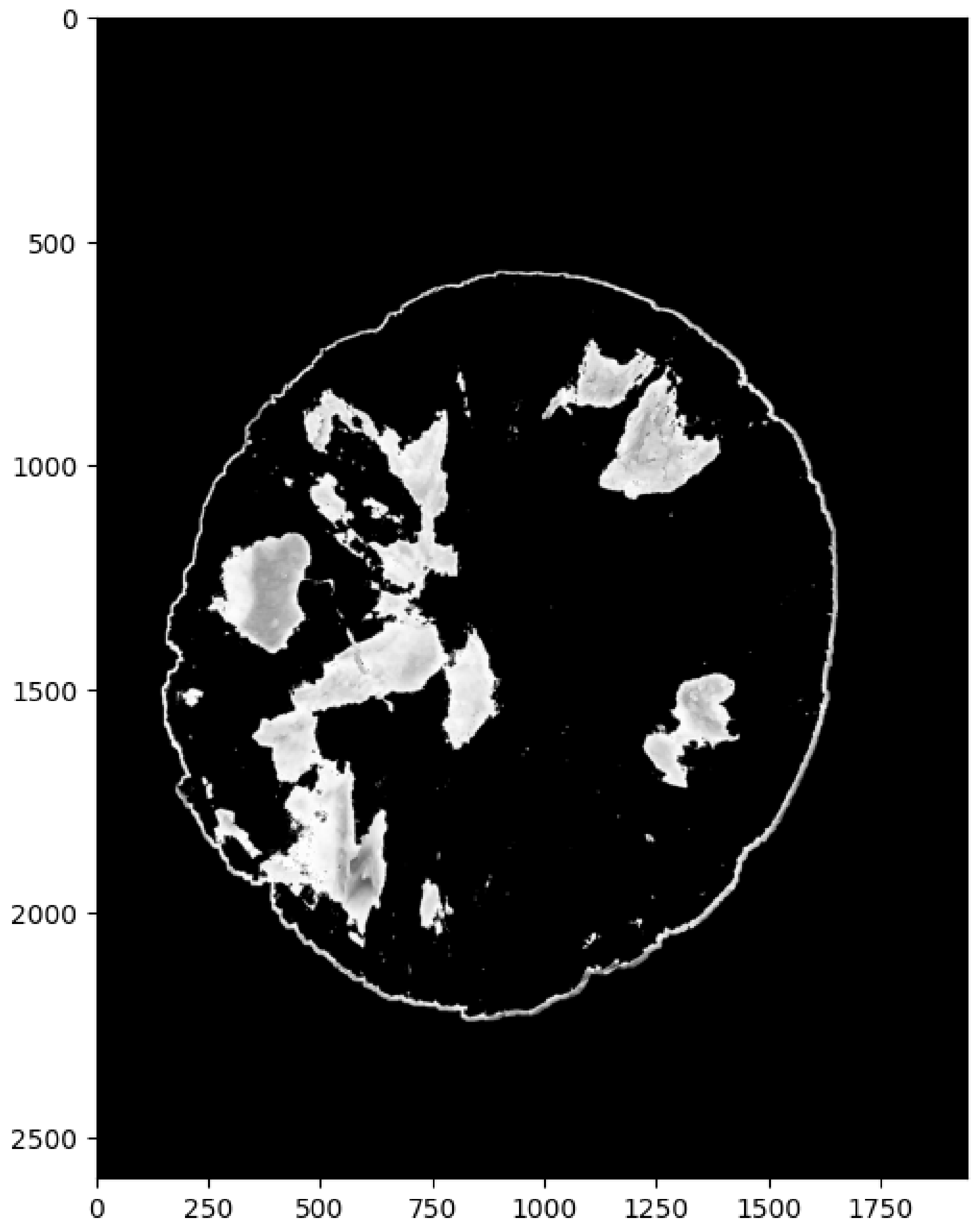

0
500
1000
1500
2000
2500
0
250
500
750
1000
1250
1500
1750

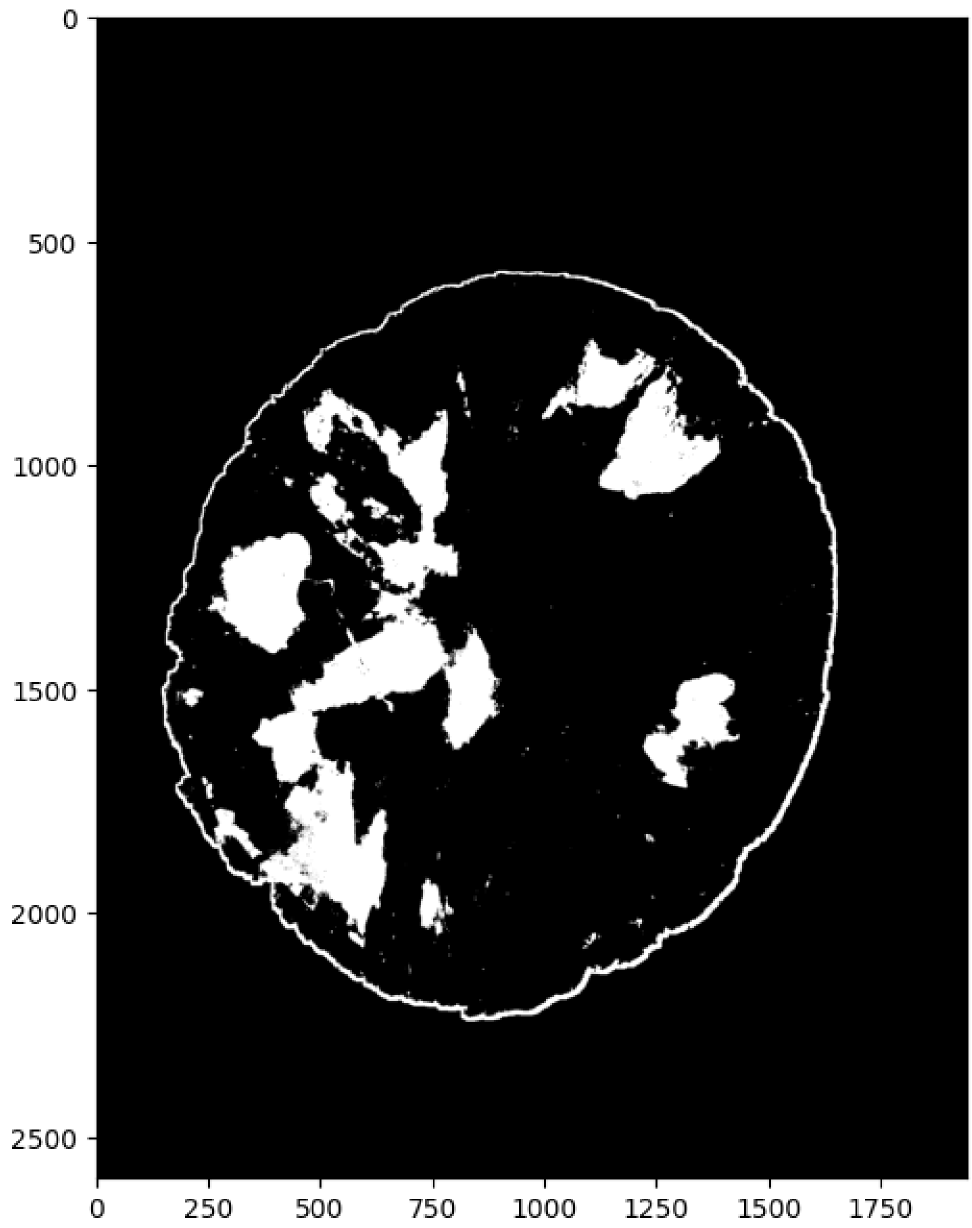

[19]:
```
# Fill in small objects/ noise
binary_img2 = pcv.median_blur(gray_img=mask3, ksize=5)

filled2 = pcv.fill(bin_img=binary_img2, size=1000)
```

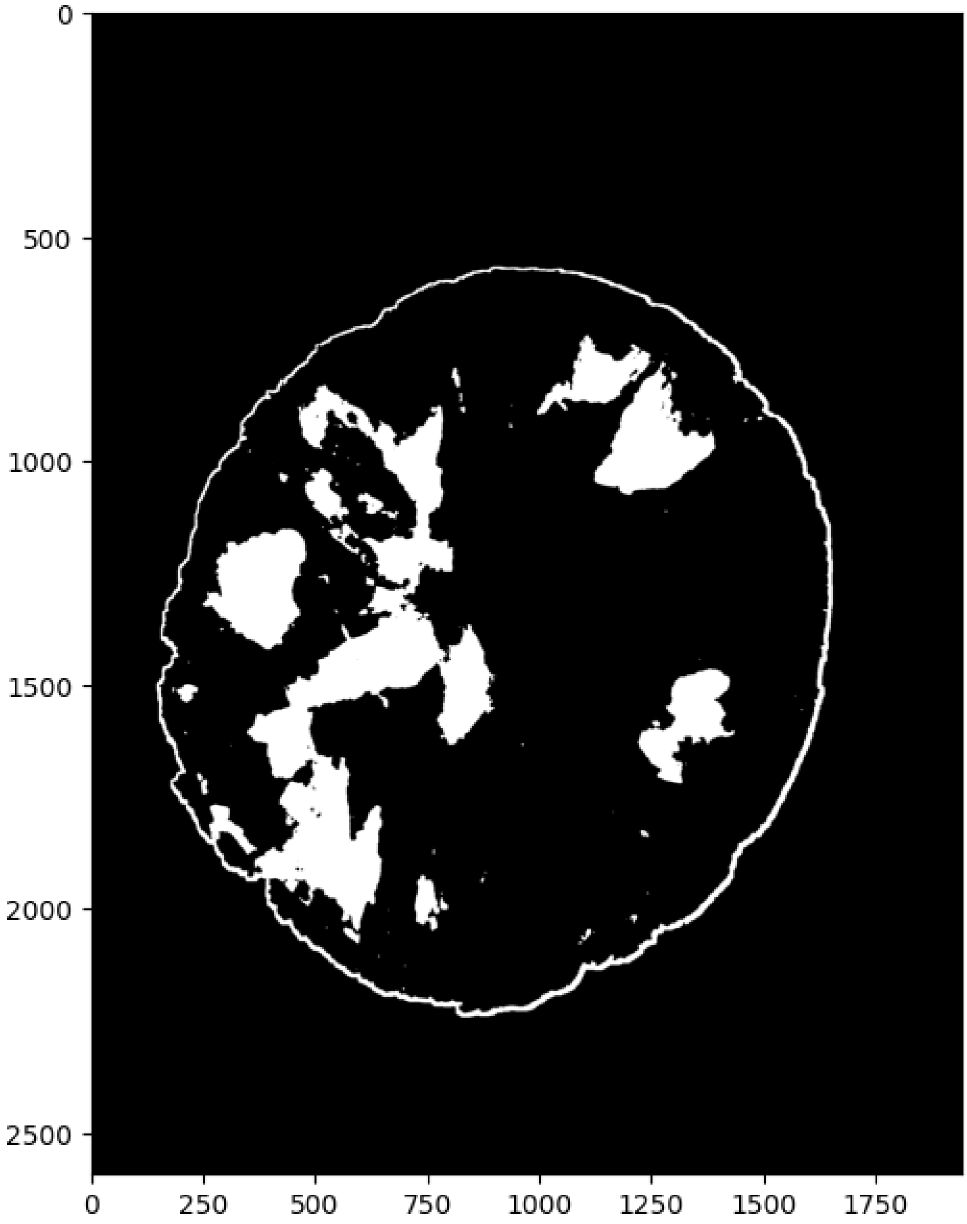
0
500
1000
1500
2000
2500
0
250
500
750
1000
1250
1500
1750

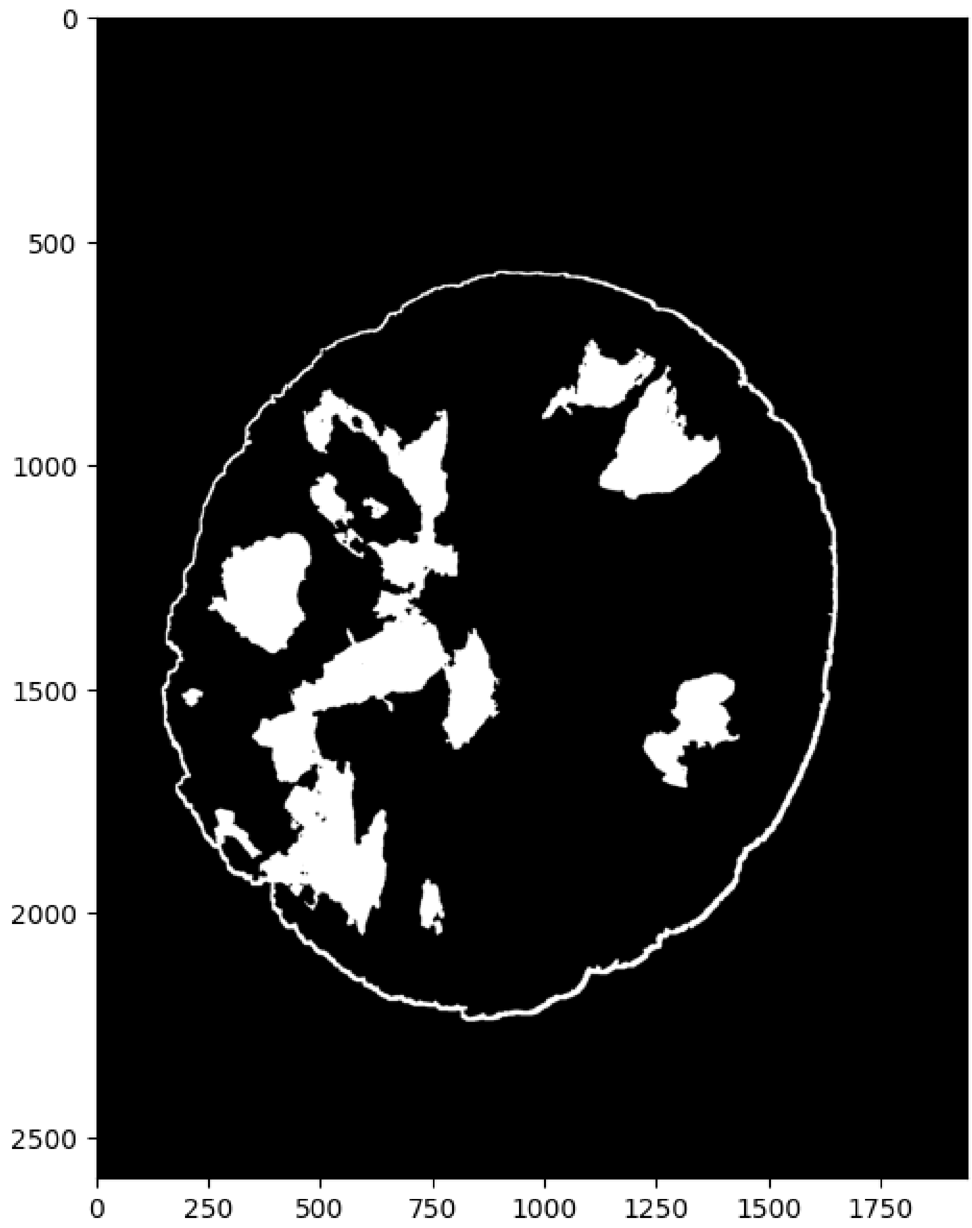

[20]:
```
# Perform erosion filtering to try and remove the outer edge of the cross section
# Results in removal of isolated pixels or boundary of object removal
er_img = pcv.erode(gray_img=filled2, ksize=4, i=3)
```

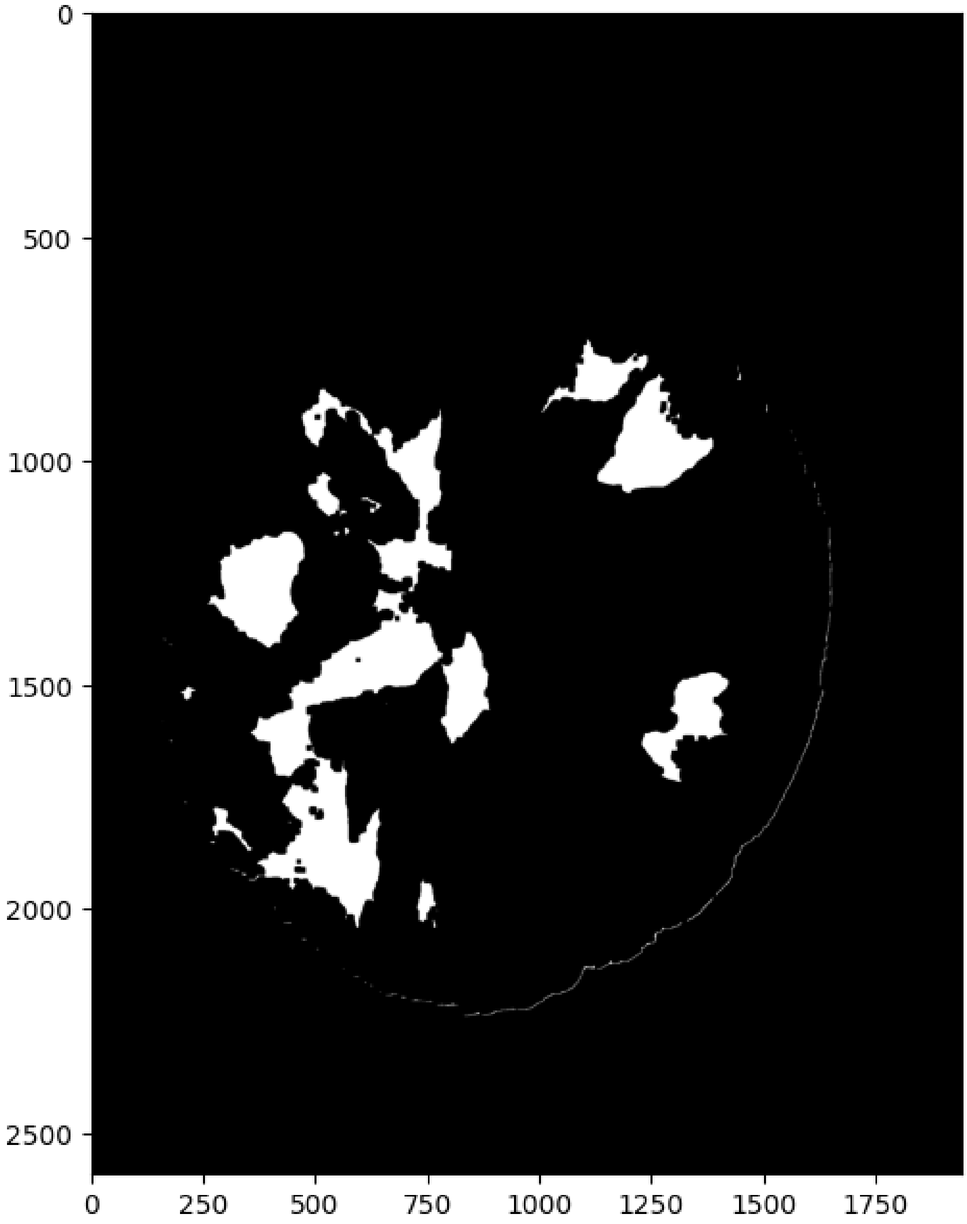

[21]:
```
#fill in small remaining specks
filled3 = pcv.fill(bin_img=er_img, size=1000)
```

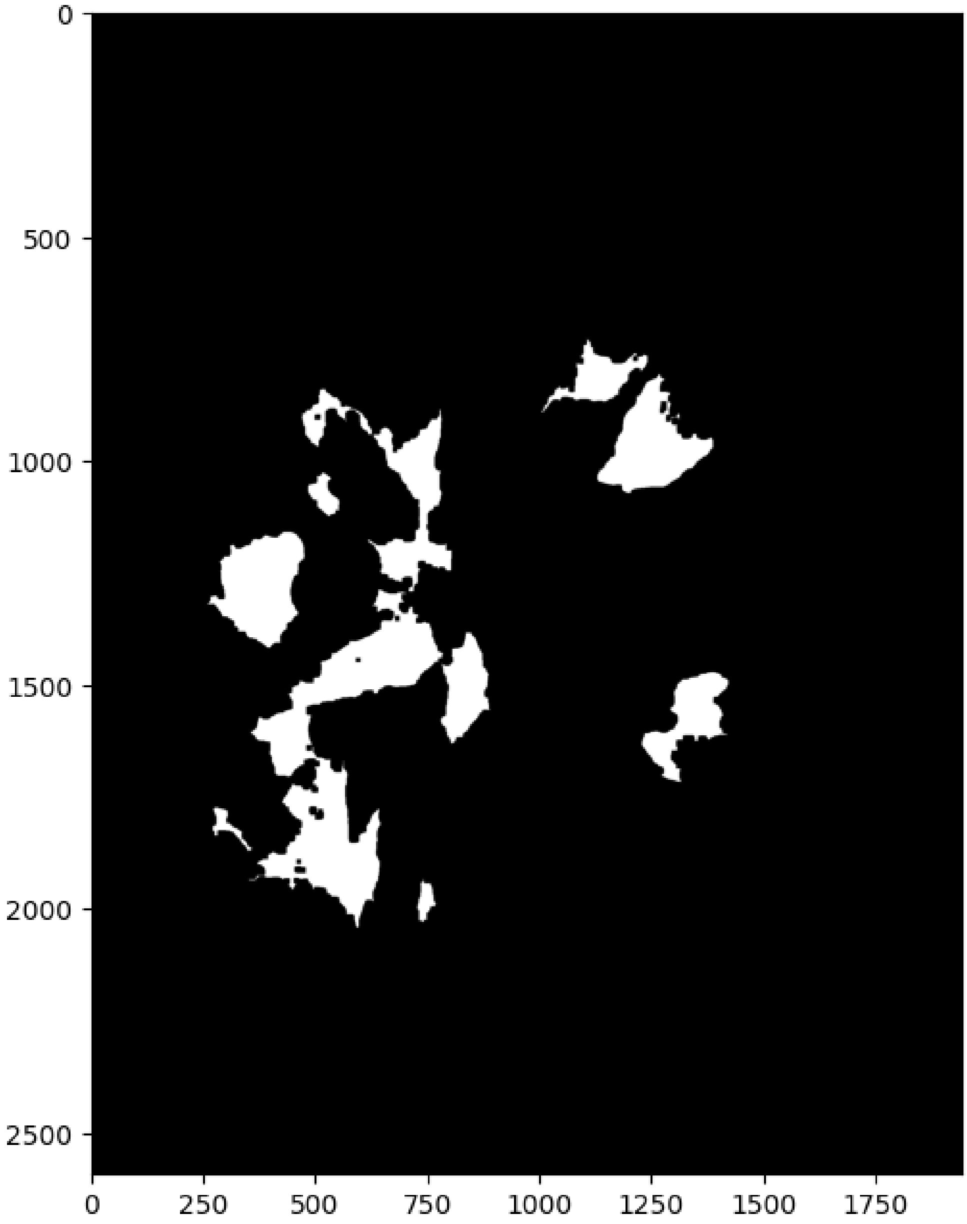

[22]:

```
#dialate to help restore original object size
dilated_necrosis = pcv.dilate(gray_img=filled3, ksize=2, i=5)
```

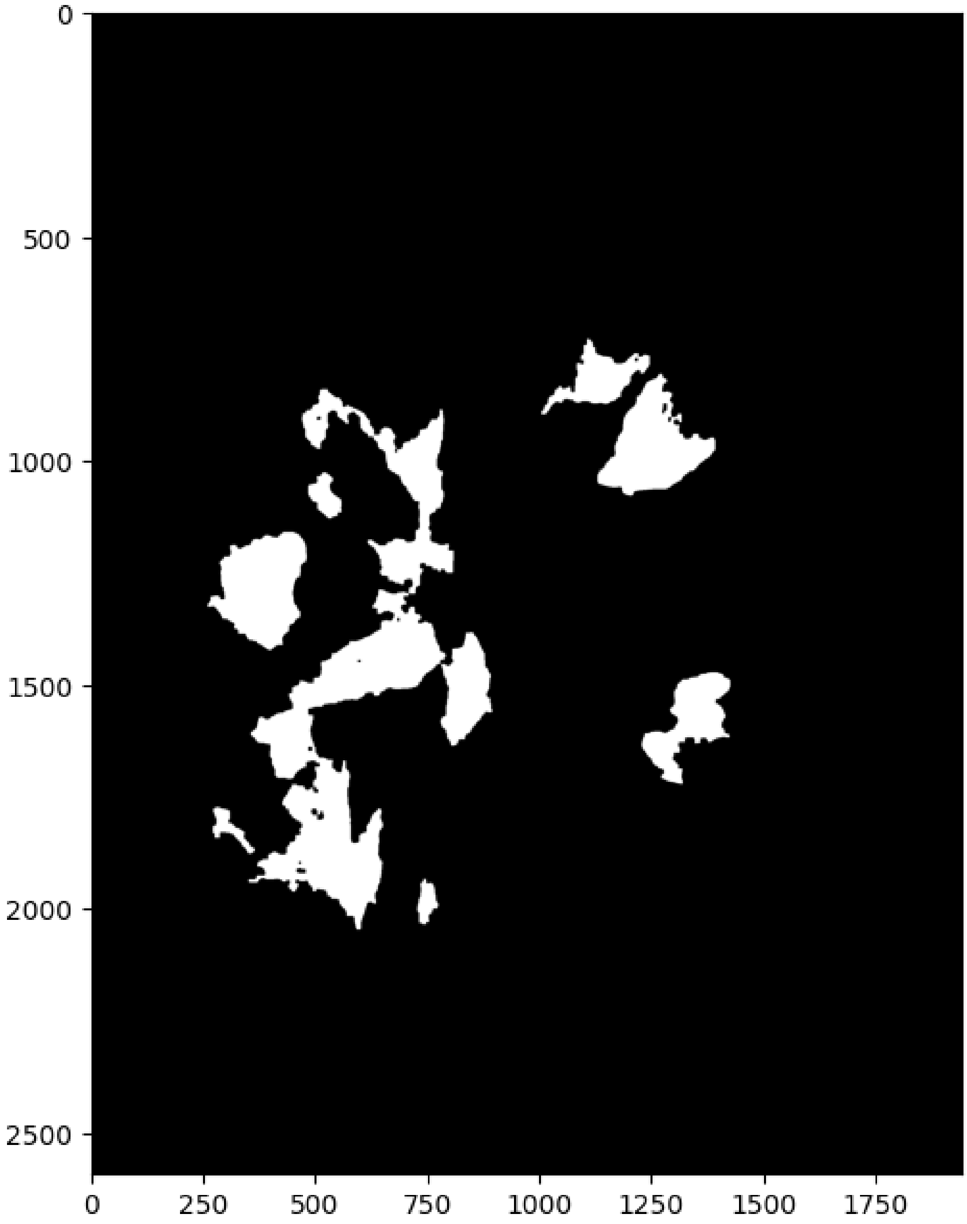

[23]:
```
# Apply mask to see what we are identifying as necrotic tissue
necrosis = pcv.apply_mask(img=masked1, mask=dilated_necrosis, mask_color='white')
```

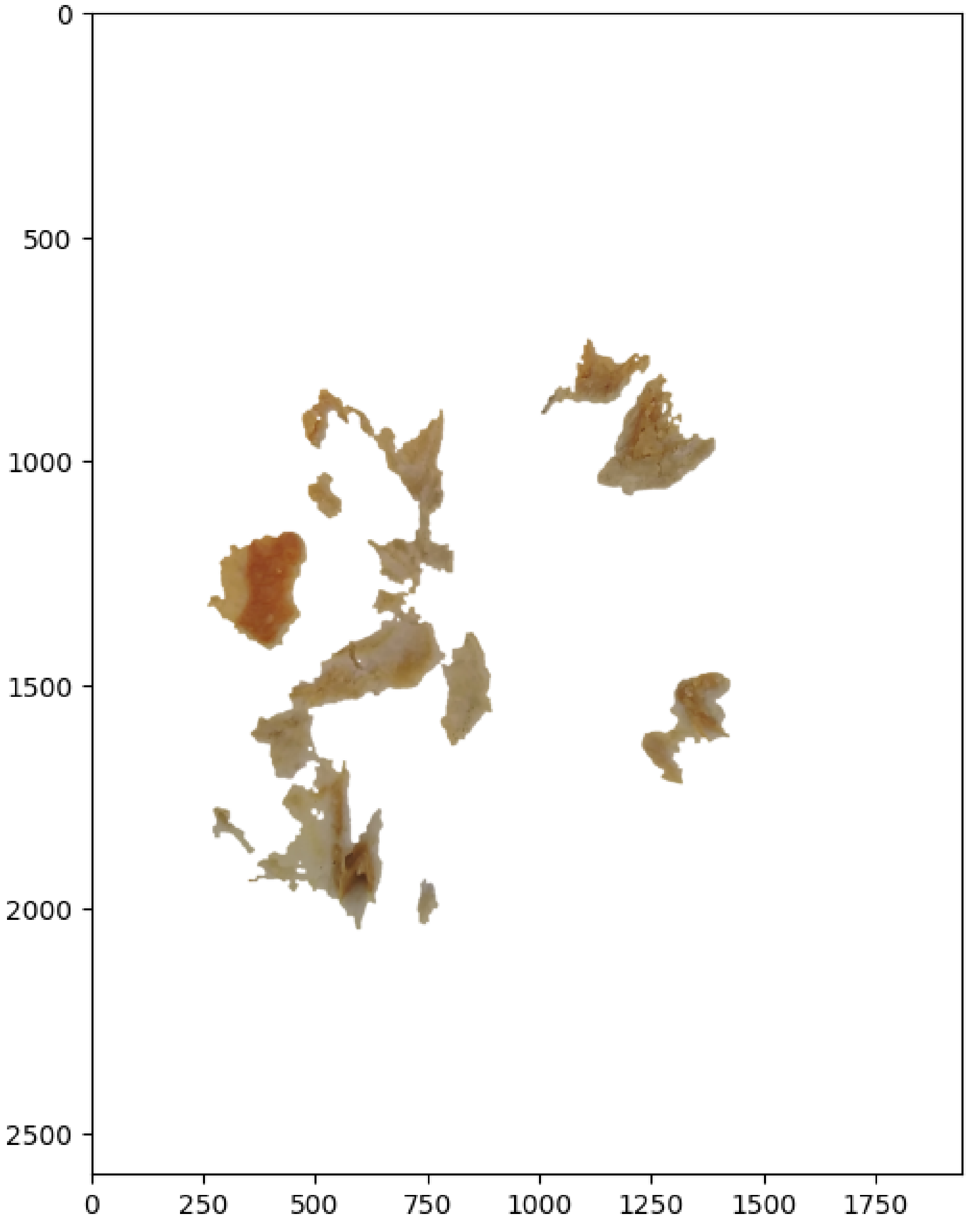

[24]:
```
# Find objects
objects2, hierarchy2 = pcv.find_objects(img=masked1, mask=dilated_necrosis)
```

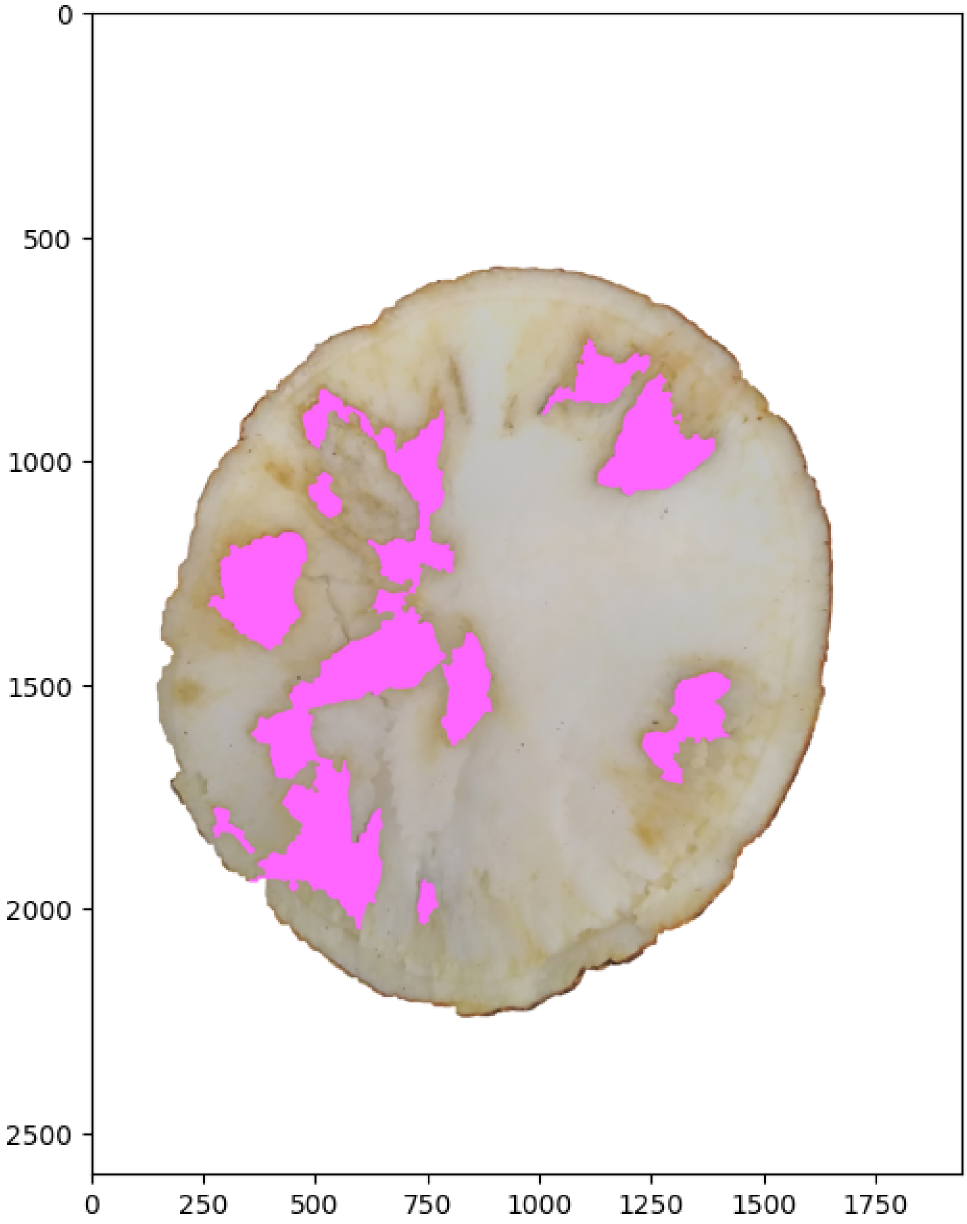

[25]:
```
#combine kept objects
obj2, mask3 = pcv.object_composition(img=img1, contours=objects2,
    hierarchy=hierarchy2)
```

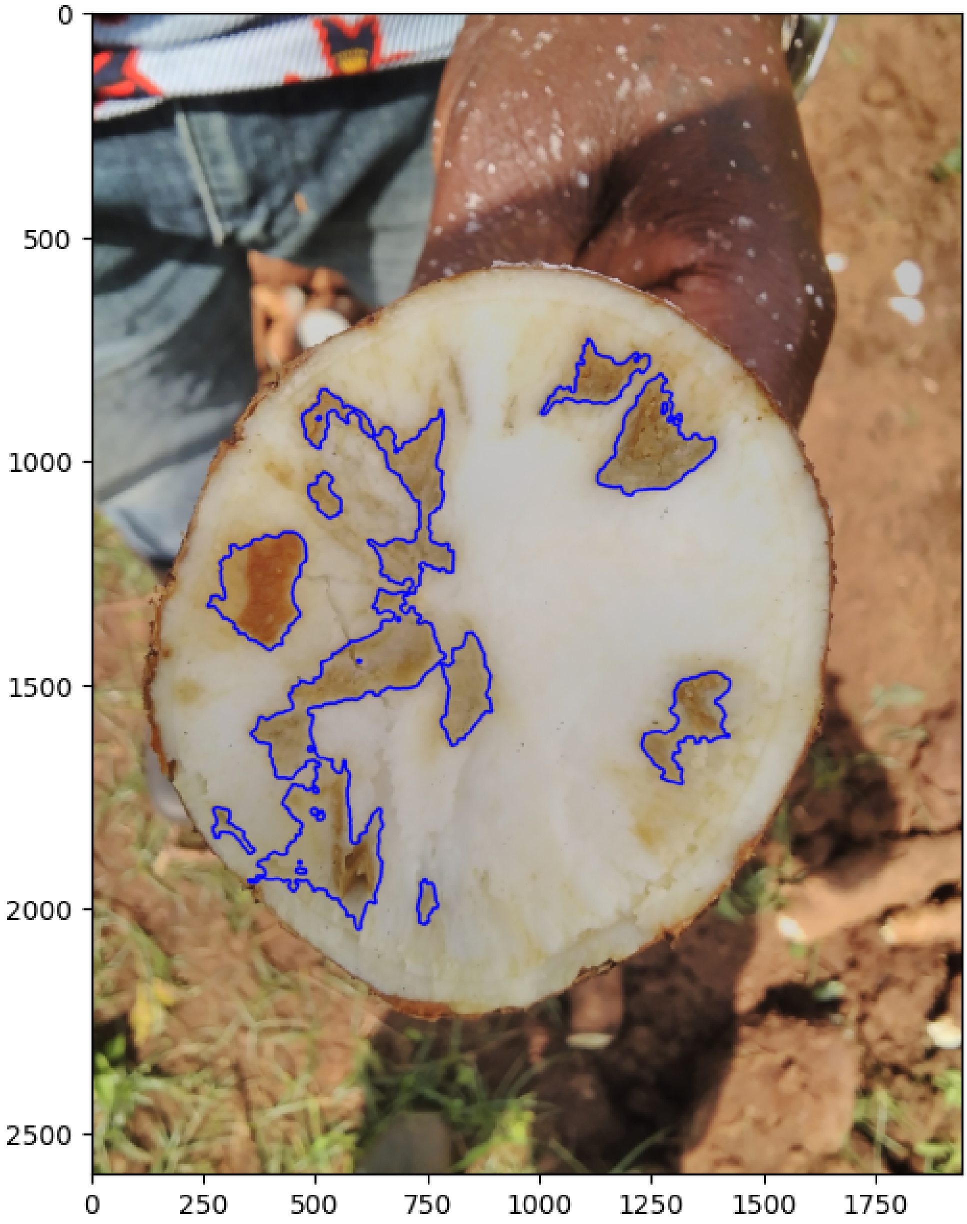
0
500
1000
1500
2000
2500
0
250
500
750
1000
1250
1500
1750

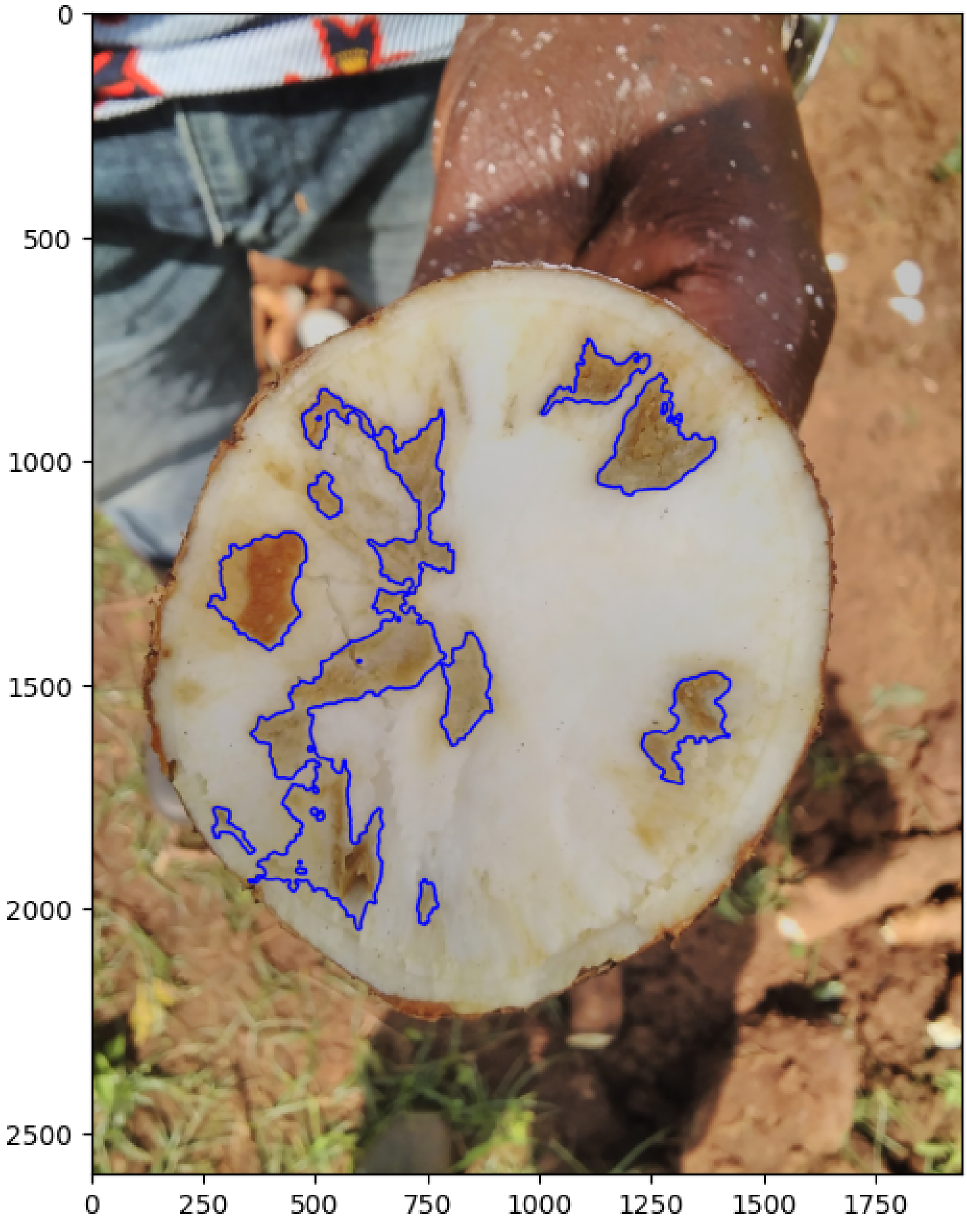


```
[26]: ############### Analysis ################

# Find shape properties of necrotic tissue, data gets stored to an outputs class␣
 ↪automatically
if obj2 is None:
    pcv.outputs.save_results(filename=args.result)

else:
```

```
   analysis_image2 = pcv.analyze_object(img=img1, obj=obj2, mask=mask3,␣
↪label="necrosis")
```

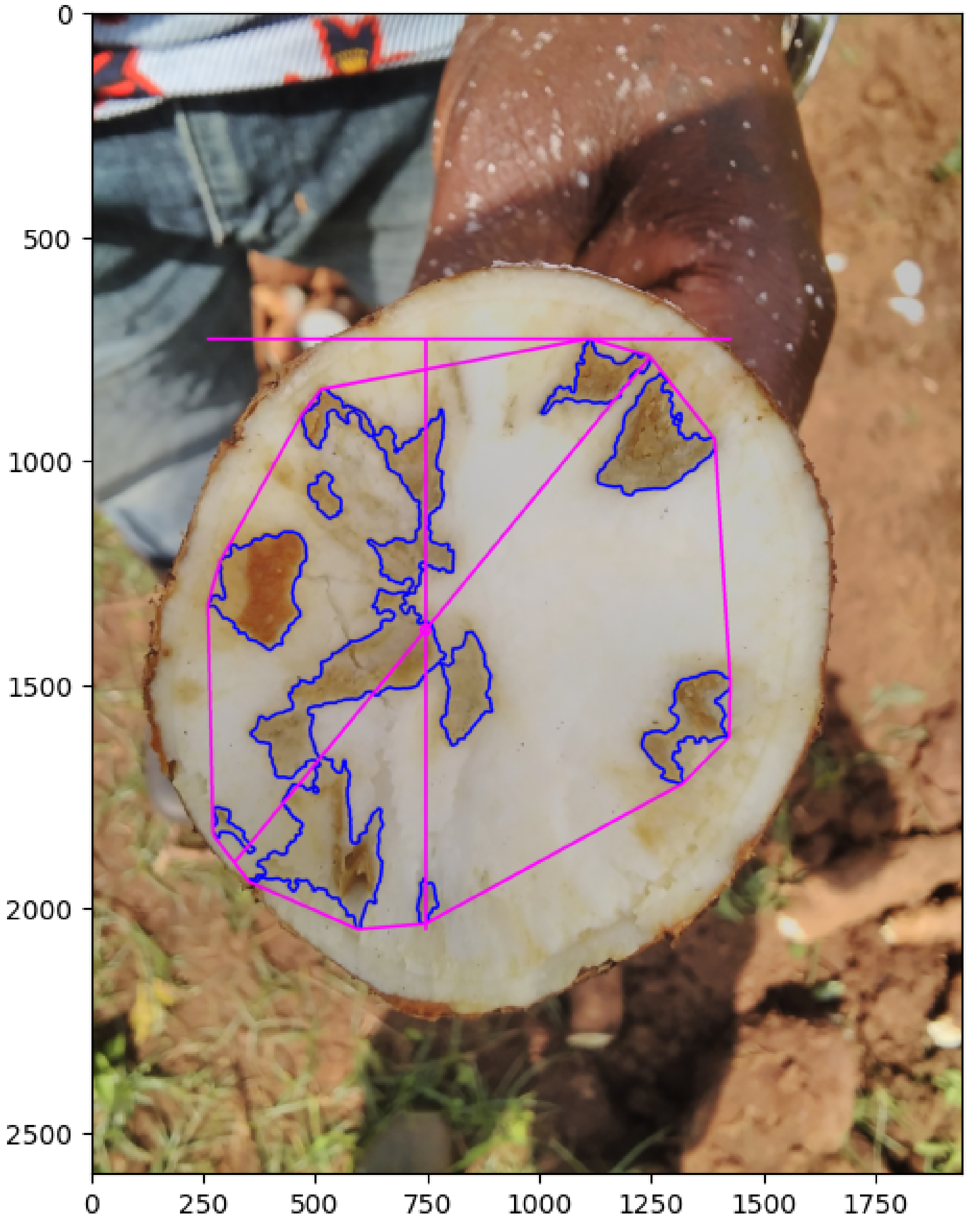


```
[27]: import numpy as np
#Calculate percent of the cross section identified as necrotic
percent_necrosis = (np.count_nonzero(dilated_necrosis)/np.
↪count_nonzero(Total_Cross_Section))*100
```

[28]:
```
# Create a new measurement ("percent necrosis") that gets saved to the outputs␣
↪class
pcv.outputs.add_observation(sample='default', variable='percent_necrosis',␣
↪trait='percent area identified as necrotic',
                            method='ratio of pixels', scale='percent',␣
↪datatype=float,
                            value=percent_necrosis, label='percent')
```

[29]:
```
#Data stored to the outputs class can be accessed using the variable name
pcv.outputs.observations['default']['percent_necrosis']['value']
```

[29]: 14.965343003676018

[60]:
```
# The save results function will take the measurements stored when running any␣
↪PlantCV analysis functions, format,
# and print an output text file for data analysis. The Outputs class stores data␣
↪whenever any of the following functions
# are ran: analyze_bound_horizontal, analyze_bound_vertical, analyze_color,␣
↪analyze_nir_intensity, analyze_object,
# fluor_fvfm, report_size_marker_area, watershed. If no functions have been run,␣
↪it will print an empty text file
pcv.outputs.save_results(filename=args.result)
```
